# Brain oscillatory activity as a biomarker of motor recovery in chronic stroke

**DOI:** 10.1002/hbm.24876

**Published:** 2019-11-28

**Authors:** Andreas M. Ray, Thiago D. C. Figueiredo, Eduardo López‐Larraz, Niels Birbaumer, Ander Ramos‐Murguialday

**Affiliations:** ^1^ Institute of Medical Psychology and Behavioral Neurobiology, University of Tübingen Tübingen Germany; ^2^ TECNALIA, Health Department Neural Engineering Laboratory San Sebastián Spain

**Keywords:** EEG, motor control, neuronal plasticity, rehabilitation, stroke

## Abstract

In the present work, we investigated the relationship of oscillatory sensorimotor brain activity to motor recovery. The neurophysiological data of 30 chronic stroke patients with severe upper‐limb paralysis are the basis of the observational study presented here. These patients underwent an intervention including movement training based on combined brain–machine interfaces and physiotherapy of several weeks recorded in a double‐blinded randomized clinical trial. We analyzed the alpha oscillations over the motor cortex of 22 of these patients employing multilevel linear predictive modeling. We identified a significant correlation between the evolution of the alpha desynchronization during rehabilitative intervention and clinical improvement. Moreover, we observed that the initial alpha desynchronization conditions its modulation during intervention: Patients showing a strong alpha desynchronization at the beginning of the training improved if they increased their alpha desynchronization. Patients showing a small alpha desynchronization at initial training stages improved if they decreased it further on both hemispheres. In all patients, a progressive shift of desynchronization toward the ipsilesional hemisphere correlates significantly with clinical improvement regardless of lesion location. The results indicate that initial alpha desynchronization might be key for stratification of patients undergoing BMI interventions and that its interhemispheric balance plays an important role in motor recovery.

## 1 INTRODUCTION

Stroke is a major global health problem. The number of stroke victims has been rising in the past years all around the world. Millions of stroke survivors are left with very limited motor function or complete paralysis and depend on assistance (Feigin et al., 2016). Therapeutic approaches such as constraint‐induced movement therapy are not applicable to the group of patients with severe limb weakness (Birbaumer, Ramos‐Murguialday, & Cohen, 2008). However, brain–machine interface (BMI) training has demonstrated efficacy in promoting motor recovery in chronic paralyzed stroke patients (Ramos‐Murguialday et al., 2013), and long term effects (Ramos‐Murguialday et al., 2019). Subsequent work has replicated and confirmed BMI efficacy. Arm and hand movements are trained using a body actuator (e.g., orthotic robots) that is controlled by oscillatory activity of the brain (Ang et al., 2014; Frolov et al., 2017; Kim, Kim, & Lee, 2016; Leeb et al., 2016; Mokienko et al., 2016; Ono et al., 2014). Brain signals can thus travel to the limb muscles along an alternative pathway. Contingently linking movement‐related patterns of brain activity and visuo‐proprioceptive feedback of the movement supports associative learning (Ramos‐Murguialday et al., 2012; Sirigu et al., 1995).

Changes in sensorimotor brain oscillations involved in planning and execution of movements were used as control signals for the BMI in the aforementioned studies. The sensorimotor rhythm (SMR) is an oscillation within the alpha frequency range of the EEG during a motionless resting state over the central‐parietal brain regions. Movement planning, imagination and execution lead to its suppression. In the present work, we investigate EEG brain oscillations of the alpha frequency, ranging from 8 to 12 Hz, over the motor cortex, and we term them “alpha oscillations.”

Biomarkers could be defined as indicators “of disease state that can be used as a measure of underlying molecular/cellular processes that may be difficult to measure directly in humans” (Boyd et al., 2017). When dealing with a condition as heterogeneous as stroke validated biomarkers of recovery could help plan treatments and support efficient allocation of resource while maximizing outcome for the patients. Alpha brain oscillations have been evaluated as markers of ischaemia and predictors of clinical outcome in acute patients (Finnigan & van Putten, 2013; Rabiller, He, Nishijima, Wong, & Liu, 2015). Desynchronization in the alpha frequency range has also been investigated as a marker of stroke and a predictor of recovery in the same patient group. Tangwiriyasakul, Verhagen, Rutten, and Putten (2014) showed that the recovery of motor function was accompanied by an increase of alpha desynchronization on the ipsilesional side. In subacute patients presenting mild to moderate motor deficits recovery lead to a similar increase of alpha desynchronization on the affected hemisphere (Platz, Kim, Engel, Kieselbach, & Mauritz, 2002). Furthermore, first attempts investigated correlations of alpha desynchronization with motor improvements in chronically impaired patients (Kaiser et al., 2012). In a controlled study, a group of subacute patients with severe deficits used motor imagery, guided by a brain–computer interface, in addition to their regular physiotherapeutic rehabilitation protocol. They showed a higher probability for motor improvements with increased alpha desynchronization (Pichiorri et al., 2015).

In the present work, we investigated what changes in the oscillatory activity of the brain a proprioceptive BMI coupled with physiotherapy produces over the course of a training intervention and if these correlate with recovery in severely paralyzed chronic stroke patients. We hypothesized that functional motor improvements are accompanied by an ipsilesional increase and a contralesional decrease in alpha desynchronization indicating reorganization of compensatory brain activity from the contralesional to the ipsilesional hemisphere. We intend to establish alpha oscillatory activity as a biomarker of motor impairment and as a building block of statistical models of stroke neurorehabilitation.

## 2 METHODS

### 2.1 Study design of the original trial

Thirty chronic stroke patients took part in the original study (Ramos‐Murguialday et al., 2013). They presented no active finger extension due to their severe motor impairment, as measured by the modified upper limb Fugl‐Meyer Assessment (FMA; Table 1). Apart from the complete paralysis of one hand, the inclusion criteria were: age between 18 and 80 years, at least 8 months since the insult, no psychiatric or neurological condition other than stroke, no cerebellar lesion or bilateral motor deficit, no epilepsy and a mini‐mental state (MMS) score of above 21. The patients were recruited publicly via stroke associations, rehabilitation centers and hospitals within Germany from December 2007 to March 2013. 504 patients were contacted, out of which 263 did not meet the inclusion criteria, 202 declined to participate and 9 were excluded because of other reasons, leading to a final pool of 30 patients. This number met the criteria for statistical power calculated in study using a similar technique (Buch et al., 2008). Half of the patients showed lesions with involvement of the motor cortex (“mixed” lesion type), the others presented subcortical lesions only (“subcortical” lesion type). The primary clinical outcome measure of the original trial was the combined modified Fugl‐Meyer assessment (cFMA). It comprises the sum of the arm and hand scores excluding scores related to coordination, speed and reflexes. The maximum score is 54 points. Details on the movements assessed in the cFMA test are presented in Supporting Information, section 5. The assessment was administered at the post test and two tests prior to the intervention. The mean of both baseline FMAs was used to calculate the difference between the values before and after the intervention.

**Table 1:** Means and standard deviations of demographic data at the time of enrollment in the study

Sex	Age (year)	Time since stroke (months)	Lesion side	cFMA scores	Lesion distribution
18 M/12 F	49.8 ± 12.4	68.5 ± 58.5	16 R/14 L	12.22 ± 8.82	Cont: 6 mixed/10 subcortical Sham: 10 mixed/4 subcortical

*Note*: The column “lesion distribution” shows the number of mixed lesions (i.e., lesions including cortical and subcortical areas) and subcortical lesions in the experimental group (“Cont”) and the control group (“Sham”).

#### 2.1.1 Standard protocol approvals, registrations, patient consent

**Figure 1:**
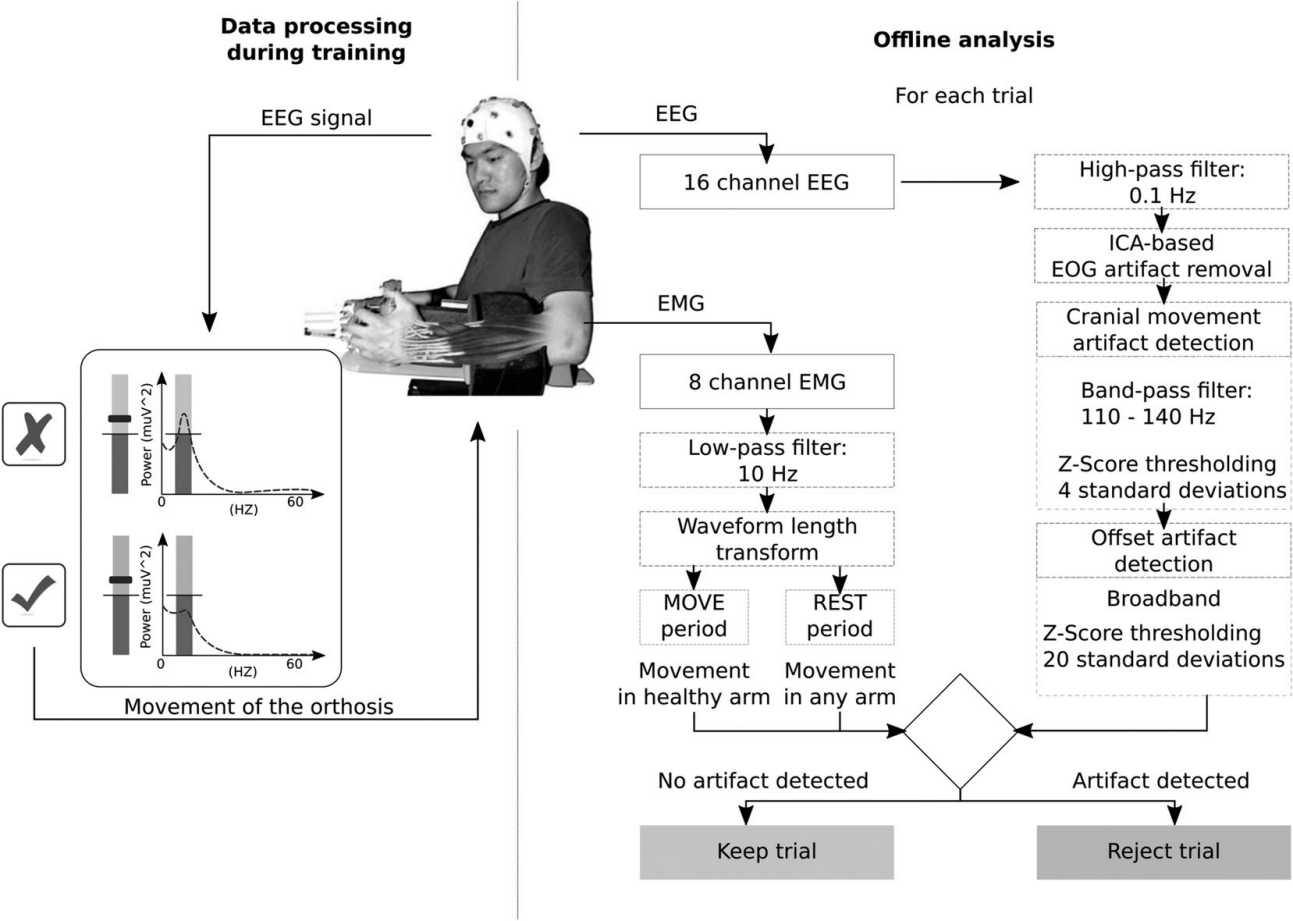
Schematics of the data acquisition phase and the offline analysis for EEG and EMG. Neurophysiological data was acquired using a 16 channel EEG cap and 4 bipolar EMG electrodes on each arm. EEG data were cleaned from eye movement artifacts and trials containing other artifacts (e.g., cranial EMG, head movements, and so on). EMG data were analyzed to detect compensatory muscle contractions on the healthy upper limb and on the paretic side during resting intervals to identify these trials as contaminated because the muscle activity is a sign of undesired EEG activity. Only data free of artifacts were used for the final analysis of EEG oscillatory activity

The original clinical trial and the analysis presented here were conducted at the University of Tübingen, Germany. Informed consent was obtained from all patients and the studies were approved by the ethics committee of the Faculty of Medicine of the University of Tübingen, Germany. Authorization has been obtained for disclosure of the person recognizable in Figure 1.

We fully acknowledge that clinical trials should be registered publicly for transparency. However, by the time the clinical trial of the original study was conducted the registration of such trials was neither mandatory nor common practice, which is why the trial was not registered. A posteriori registration of the trial is pending.

#### 2.1.2 Intervention protocol of the original trial

The patients were randomly divided into an experimental group (*n* = 16) and a control group (*n* = 14). The original study was double‐blinded to avoid potential bias introduced by experimenters. In both groups, electric brain activity was recorded using electroencephalography (EEG). Changes in the SMR of the ipsilesional hemisphere during movement attempts of fingers and arm were contingently translated into movement of the arm and hand orthosis only in the experimental group. Decrease of the power of the SMR with respect to baseline led to movement of the arm or the hand and a relative increase stopped the movement. In the control group, the setup was similar but the movements executed by the robot were independent of brain activity. The movements were triggered randomly but the period of time the orthosis was moving was approximately equivalent to that of the experimental group. Both groups received identical physiotherapy after the BMI training. Each subject performed 17 ± 1.8 (mean ± *SD*) sessions of BMI‐training within a period of up to 6 weeks. Each session consisted of 165 ± 19.5 (mean ± *SD*) trials. A training trial consisted of an intertrial interval (4–7 s), a preparation phase (2 s) and the movement phase (5 s). The reader is kindly referred to the original article for more details on the intervention protocol. Lesion maps obtained by magnetic resonance imaging are presented in Supporting Information of the original article (Ramos‐Murguialday et al., 2013).

#### 2.1.3 Neurophysiological recordings

EEG data were recorded using a 16‐channel ActiCap from locations Fp1, Fp2, F3, Fz, F4, T7, C3, Cz, C4, T8, Cp3, Cp4, P3, Pz, P4, and Oz and a BrainAmp 32‐channel amplifier (Brainproducts GmbH, Munich, Germany). The ground electrode was placed at AFz and the reference at FCz. Furthermore, EOG electrodes for detection of vertical and horizontal eye movements were used and surface electromyography electrodes (EMG) were fixed to four muscle groups on both upper limbs (extensor carpi ulnaris, extensor digitorum, long head of the biceps, external head of the triceps) in order to monitor movement onsets or involuntary contractions. The signals were sampled at 500 Hz. The arm orthosis was a ReoGo rehabilitation robot (Motorika, Cesarea, Israel). We used a robotic orthotic system developed in‐house to exert hand movements.

The individual SMR frequency was obtained from EEG recorded in a calibration session on the day before the training. The power of the EEG signal while the patients rested and while they were trying to open and close the paretic hand was compared. The frequency range showing the maximum variance between the two conditions as measured by the coefficient of determination *r*^2^ was defined as individual SMR frequency. The most discriminative electrodes in the central‐parietal region were selected.

### 2.2 Artifact detection

Even though the participants were instructed to minimize movements of head and body during the recordings, contamination of the EEG by movement artifacts could not be completely prevented as the experiment involved movements of the body. Detection and rejection of artifacts in the data were carried out using a fully automated process (Figure 1). First, artifacts caused by eye movements were removed from the EEG signal by way of extracting the independent components representing these artifacts identified in the EOG (Halder et al., 2007). Then, trials contaminated by cranial muscle artifacts were detected. The EEG signal of all channels was filtered between 110 and 140 Hz and the signal in each channel was *z*‐scored and the *z*‐values were averaged per sample. A threshold of 4 *SD*s was applied to remove trials containing artifacts. Afterward, a similar procedure was performed on the broadband EEG signal with a threshold of 20 standard deviations to remove trials containing offset artifacts. The fieldtrip toolbox was used for rejection of EEG artifacts (Oostenveld, Fries, Maris, & Schoffelen, 2011). Finally, the EMG activity was analyzed. The waveform length of the EMG was computed (Ramos‐Murguialday, Soares, & Birbaumer, 2010). Muscle contractions were identified by the Waveform length exceeding 3 *SD*s of the data. Any such arm or hand movement during the rest period or movements of the healthy limb during the phase of the movement attempt led to removal of the trial from the analysis.A session was excluded from the analysis if less than 10% (16 trials) of all trials remained. If half of the total number of sessions of a patient were removed, the subject was excluded from the analysis. One patient was considered an influential outlier in the statistical modeling due to consistent undesired synchronization of central alpha brain oscillations during movement phases of the training in most sessions. This patient was thus excluded from the analysis. The rigorous rejection procedure led to a final pool of 22 subjects. This conservative procedure facilitates interpretability of the results. Descriptive statistics on the rejection of trials and an overview of the number of trials, sessions and subjects removed is presented in Supporting Information, section 1.

### 2.3 Movement‐related features of the EEG power spectrum

Movement planning, imagination, and execution lead to suppression of brain oscillatory activity over the motor cortex. It has been shown that stroke patients can (re‐) learn to voluntarily modulate this rhythm to control movements of their paretic limbs by way of robotic orthoses (Buch et al., 2008; Ramos‐Murguialday et al., 2013). The phenomenon is often defined as mμ rhythm or as SMR. There are various works describing the effect within the alpha frequency range of the EEG (Klimesch, Sauseng, & Hanslmayr, 2007; Kuhlman, 1978; Pfurtscheller & Lopes da Silva, 1999). Similar synchronization and desynchronization effects have been reported with other functional relevance in the beta frequency range (van Wijk, Beek, & Daffertshofer, 2012). Peak frequency and amplitude of the SMR vary between individuals but movement‐related desynchronization in healthy populations spreads across the whole alpha range (Pfurtscheller, 2003). As alpha oscillations may also constitute indicators of underlying processes not related to movements it may be difficult to discern alpha central oscillations from genuine sensorimotor oscillatory activity in patients involved in visuo‐proprioceptive motor tasks (Klimesch et al., 2007). However, since first, significant power decreases in healthy subjects during execution of a BMI lasting several seconds have mainly been found in the alpha frequency range (Ramos‐Murguialday & Birbaumer, 2015) and second, the SMR frequency (defined as the frequency range with the largest difference between movement attempts of the paralyzed limb and resting state) in the original trial, were also centered in the alpha frequency band (mean 10.6 Hz ± 4.8) we focused our analysis on the progression of alpha desynchronization. Furthermore, to disentangle effects of individualized SMR values used for BMI intervention from the general alpha band, we also evaluated the progression of desynchronization in the individual SMR frequency. More information can be found in Supporting Information, section 4.

Nevertheless, recent work has identified beta oscillations as potential therapeutic target for stroke rehabilitation because these oscillations are involved in cortical disinhibition and have been suggested as the rhythm connecting brain and muscles (Mima, Toma, Koshy, & Hallett, 2001; Naros & Gharabaghi, 2015; Rossiter, Boudrias, & Ward, 2014). Therefore, we also analyzed the progression of desynchronization in the beta frequency band (12–25 Hz). More information can be found in Supporting Information, section 4.

A previous work of our group on a similar dataset involving movement attempts in chronic stroke showed the adverse influence of low frequency (1–4) and high frequency (30–48 Hz, that is, gamma band) artifacts on time‐frequency analysis of movement‐related desynchronization and classification of EEG signals (López‐Larraz et al., 2018). Therefore, gamma oscillations (30–48 Hz) were not considered in the present analysis.

Event‐related desynchronisation (ERD) was calculated following Pfurtscheller and colleagues (Pfurtscheller & Lopes da Silva, 1999) as the proportional decrease of EEG power in a movement attempt interval, M, relative to a reference interval, *R*:

(1) $$\mathrm{ERD}=\frac{M-R}{R}\times 100\%$$

ERD over the sensorimotor cortex was extracted from both hemispheres separately using the EEG signal of the electrodes C3, Cp3, P3 and C4, Cp4, P4, respectively, and within the alpha frequency band (8–12 Hz). The power spectral density was computed using Welch's method and the mean power of that frequency range was extracted. Furthermore, the EEG power was averaged over the three channels on each hemisphere. Note that because of averaging the power of the three channels no other spatial filters were used. Mean ERD was computed as described in Equation (1) over all trials of each session using the EEG data of the last 4 s of the intertrial interval as reference *R* and the EEG data of the movement attempt phase as *M*.

It is important to note that a larger relative difference between neural activity during rest (synchronized, larger EEG power) and action (desynchronized, smaller EEG power) is represented by a numerically smaller, more negative ERD value (Equation 1) and vice‐versa. We thus report “strong” ERD when the ERD values are more negative and “weak” ERD when they are less negative.

Works on brain oscillatory biomarkers of stroke rehabilitation were often limited to predicting behavioral changes by brain activity measured before and after spontaneous recovery or intervention (Stinear, 2017). Here, a comparison of ERD during movement attempts of the upper limb without the afferent input of the orthosis before and after the intervention indeed did not reveal a generalized change of ERD. For each patient a premeasurement and a post measurement involving movement attempts of the paretic arm without the orthosis were performed. The patients were asked to perform up to 85 repetitions of 3 s of resting and 4 s of movement attempts. The EEG data was preprocessed and the mean ERD of each patient before and after the intervention (pre and post) was computed. A difference of the ERD values between groups and time points could not be found (see Supporting Information, section 3). In the present work, however, we make use of the large amount of longitudinal neurophysiological data gathered during dozens of training sessions to infer on the relationship of progression of changes of brain activity and behavioral improvements. All the analysis was performed using EEG and EMG data acquired during the interventional sessions, in which the patients tried to move their paretic limb avoiding compensatory movements and the limb moved according to the brain‐controlled robotic orthosis. During the intervention proprioceptive feedback usually lead to an increased SMR desynchronization.

### 2.4 Statistical modeling

In order to model the cross‐sectional response (the clinical outcome measure ΔcFMA) with the longitudinal predictors (progression of the ERD across training sessions) we employed a two‐stage modeling process. First, the individual time courses of the ERD of all patients were modeled using a linear mixed‐effects model. In the second step, the coefficients of these modeled time courses were used to predict each patients' motor improvement.

Linear mixed‐effects models are suited for describing longitudinal physiological data because (a) they allow to reflect individual differences of intercepts and slopes with respect to population means; (b) data may be modeled even though measurements are unequally timed; (c) the number of measurements per subject is not required to be equal (Lang et al., 2016) (for a thorough description of linear mixed models, LMEMs, see [Verbeke & Molenbergs, 2001]). Shetty, Morrell, and Najjar (2009) showed that estimating the value of the explanatory variable(s) with a LMEM approach leads to the best regression parameters for predicting a clinical outcome.

Using this approach, in the first step of modeling a LMEM is constructed to estimate two coefficients per patient which describe the initial state and the progression of the ERD of each patient throughout the course of the intervention. The response variable (ERD) is thus modeled by a general intercept (representing the mean initial ERD value of all patients) and the general change over time (mean unit change of the ERD per BCI‐training session of all patients) as fixed effects. Both intercept and change over time may vary for each patient, and are therefore also introduced as random effects in the LMEM. The model thus yields two coefficients per patient: the individual progression of the ERD over time (the individual model slope), and the subject‐specific initial value of the ERD (the individual model intercept). In the second step of the procedure a linear regression model (LM) is constructed that predicts the change of the clinical outcome (ΔcFMA) by the patients' individual dynamics of the ERD, which are represented by the coefficients modeled in the first step of the procedure. From this model an inference can be made if and how the initial state of the ERD of each patient, the progression of the ERD throughout the training and the interaction between these two factors predict the motor improvement. The flow‐chart in Supporting Information, section 2 provides an intuitive description of the modeling procedure.

To assess the interhemispheric asymmetry of brain activation during recovery, the laterality coefficient is often used (Kaiser et al., 2012; Pivik, Broughton, Davidson, Fox, & Nuwer, 1993; Tangwiriyasakul et al., 2014; van Putten, 2007). The sign of the coefficient represents the laterality of the desynchronization, that is, which of the hemispheres is more active during a certain condition such as the movement of the paretic arm. In order to assess the progression of the asymmetry of the interhemispheric oscillatory activity of the brain, we expanded the laterality coefficient to encompass the temporal component (training progression). The progressive laterality coefficient (pLC) is computed as:

$${\mathrm{pLC}}_{\mathrm{ERD}}=S_{H}-S_{L}$$

The change (i.e., slope) of the ERD for each patient, was extracted from the LMEM from the data of both hemispheres (healthy hemisphere: *S*_*H*_, hemisphere of the lesion: *S*_*L*_) and subtracted from each other to form the pLC. This measure describes the progression of the asymmetry of the desynchronization between both hemispheres over the course of the training. It may reveal if the change of desynchronization throughout the intervention was stronger on one hemisphere than on the other. The values of the *pLC*_*ERD*_ were correlated with the primary clinical outcome ΔcFMA to investigate the relevance of progressive brain activity asymmetry for motor improvement.

## 3 RESULTS

### 3.1 Prediction of ΔcFMA from contralateral/ipsilesional EEG changes

The linear models for the ERD in the alpha frequency range were constructed, each predicting ΔcFMA using the coefficients extracted from the corresponding linear mixed effects model: the progression of the ERD throughout the intervention sessions and the initial ERD magnitude. An interaction term was included in the LM to investigate if the initial ERD modulated the progression of the oscillatory activity. An *F*‐test of the regression equation was significant: *F*(3, 18) = 6.96, *p* = .0026 and an adjusted *r*^2^ = .46.

**Figure 2:**
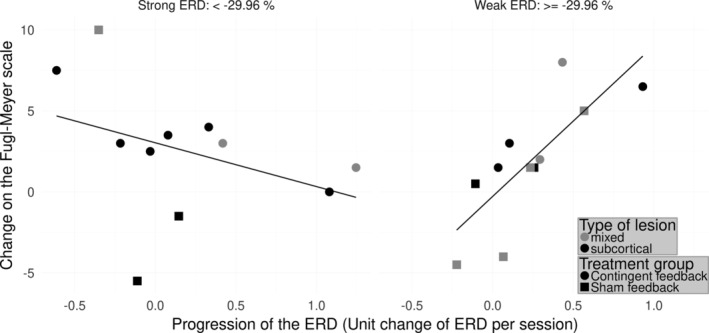
Linear model predicting the improvement of motor function (ΔcFMA) on the hemisphere of the lesion. Linear model predicting the improvement of motor function (ΔcFMA) by the initial ERD and the progression of the ERD of the alpha frequency range on the ipsilesional hemisphere over sessions: Adjusted *r* ^2^ = 0.46; *F*(3, 18) = 6.96, *p* = .0026. For improved visualization of the effects of both explanatory variables in the model the patients are separated into two cross‐sections showing relatively strong ERD (left panel) and a second group showing relatively weak initial ERD (panel on the right).For the patients showing strong ERD the inverse linear relationship of the variables suggests that the more these patients increase their ERD the larger the improvement. For the patients showing a relatively weaker ERD at the beginning of the training, the opposite relationship is apparent

In linear models with interaction terms two independent variables might exert an effect on the dependent variable. They might also modulate each other. In order to understand and interpret the interaction the data is usually separated into smaller subsets (Aiken & West, 1991). One variable is “fixed” and defines these subsets while the other variable is investigated independently within each subset. This procedure allows to observe whether the value of the “fixed” variable influences the “free” variable depending on the subset or not. If the “fixed” variable is categorical the subsets are naturally defined. However, here, the variable of interest, initial ERD, is a continuous variable and the separation is defined based on prior knowledge and the characteristics of the data (Aiken & West, 1991). Given the amount of data points a division into few subsets is the best choice. Furthermore, even though there is no standardized definition, “strong initial ERD” and “weak initial ERD” may be meaningful for the interpretation. For these reasons, we split the data into equal subsets at the median. The procedure supports intuitive visualization of the linear model (Breheny & Burchett, 2016). Moreover, it facilitates interpretation of the analysis of the brain activity on the healthy hemisphere because we saw that the ipsilesional brain activity of the patients is modulated differently in the subgroups. We thus show the correlation of the progression of ERD and ΔcFMA for two subgroups presenting relatively strong and relatively weak initial ERD (higher and lower than the median). The median value of the initial ERD is Median_*α*_ =  − 29.96% (Figure 2). Those patients presenting a relatively strong ERD at the beginning of the intervention (Figure 2, panel on the left) improved if their ERD progressively increased throughout the training. In contrast, those patients whose ERD was already relatively weak at the beginning of the intervention (Figure 2, panel on the right) improved if their ERD progressively decreased throughout the training. These relationships are also reflected in visualization of time‐frequency representations (cf., Supporting Information, section 7). It is noteworthy that four patients of the control group presented a negative change of their cFMA score regardless of their ERD progression throughout the intervention (two squares below the zero line in Figure 2).

The progression of the ERD in the beta frequency range (12–25 Hz) and the individual SMR frequency were also analyzed in the same way as the data of the alpha frequency range to complement the analysis. The results and plots are presented in Supporting Information, section 4. In summary, the *F*‐test of the regression equation of the model of the beta band was not significant. The linear model for the individual SMR frequency was significant (*F*[3,18] = 3.475, *p* = .038). The fit was lower than that of the model for alpha: *r* ^2^ = .26.

### 3.2 Prediction of ΔcFMA from ipsilateral/contralesional EEG

**Figure 3:**
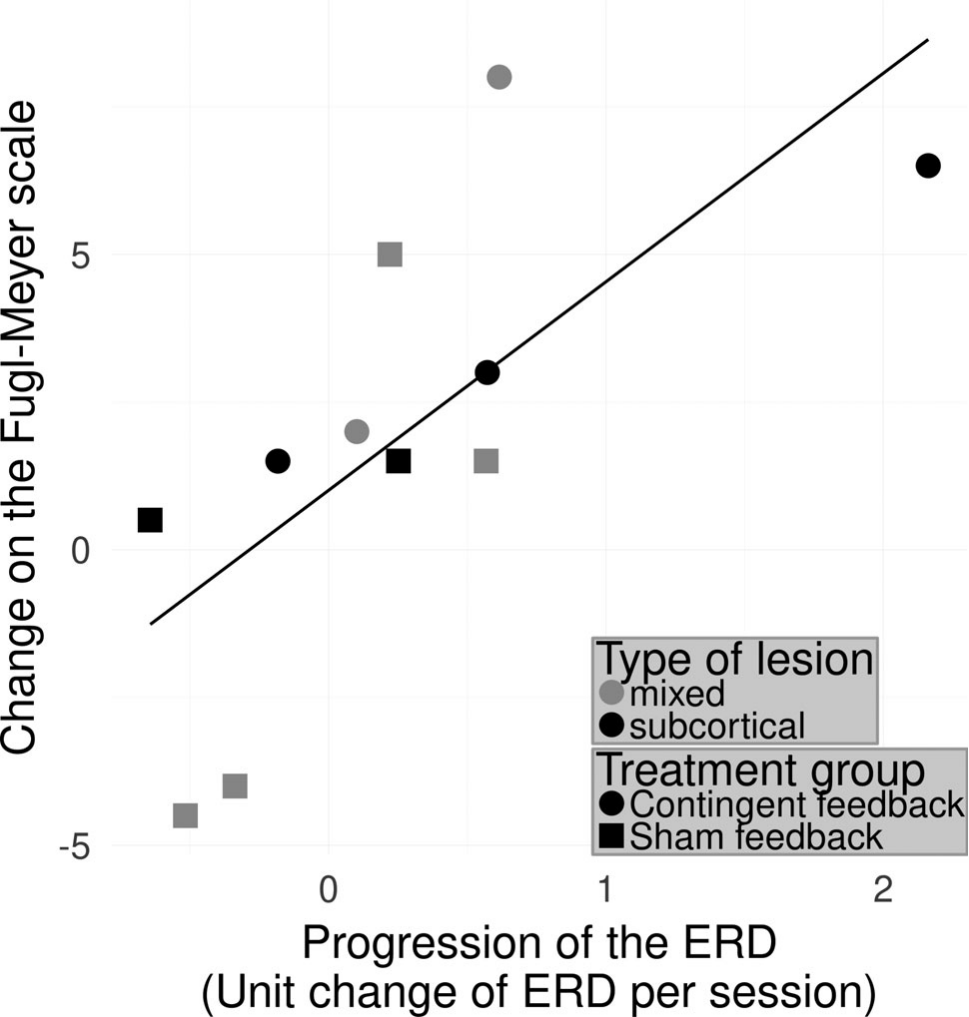
Linear model predicting the improvement of motor function (ΔcFMA) on the healthy hemisphere. Linear model predicting the improvement of motor function (ΔcFMA) by the progression of the ERD of the alpha frequency range on the healthy hemisphere over all sessions for the patients showing relatively weak initial ERD on the ipsilesional hemisphere: Adjusted *r* ^2^ = 0.45; *F*(1,9) = 9.05, *p* = .015. Better recovery was achieved when the ERD on the healthy hemisphere decreased in the course of the training

We examined how the progression of the ERD of the healthy hemisphere relates to the clinical improvement depending on the initial ERD of the lesioned hemisphere. Knowing that the initial ERD on the ipsilesional hemisphere interacts with the ERD progression patients were again separated into the same two subgroups accordingly (relatively strong and relatively weak ipsilesional ERD). The progression of the ERD on the healthy hemisphere during movements of the paretic arm and hand were modeled for both subgroups. These linear models predicted the change of the clinical outcome measure ΔcFMA. For the subgroup showing relatively weak ERD at the beginning of the intervention on the ipsilesional hemisphere the model showed a significant positive linear relationship: Adjusted *r*^2^ = 0.47; *F*(1,9) = 9.05, *p* = .0148. For the other subgroup, the patients showing relatively strong initial ERD, however, the *F*‐test for the regression equation was not significant: Adjusted *r*^2^ = −0.11; *F*(1,9) = 0.0072, *p* = .93 (Figure 3).

In summary, the patients presenting a relatively weak ipsilesional ERD at the beginning of the intervention, presented a larger motor improvement if their ERD decreased on the healthy hemisphere (i.e., activating their contralesional hemisphere less) during paretic hand movements using the BMI.

### 3.3 Prediction of ΔcFMA from interhemispheric asymmetry of brain activation

**Figure 4:**
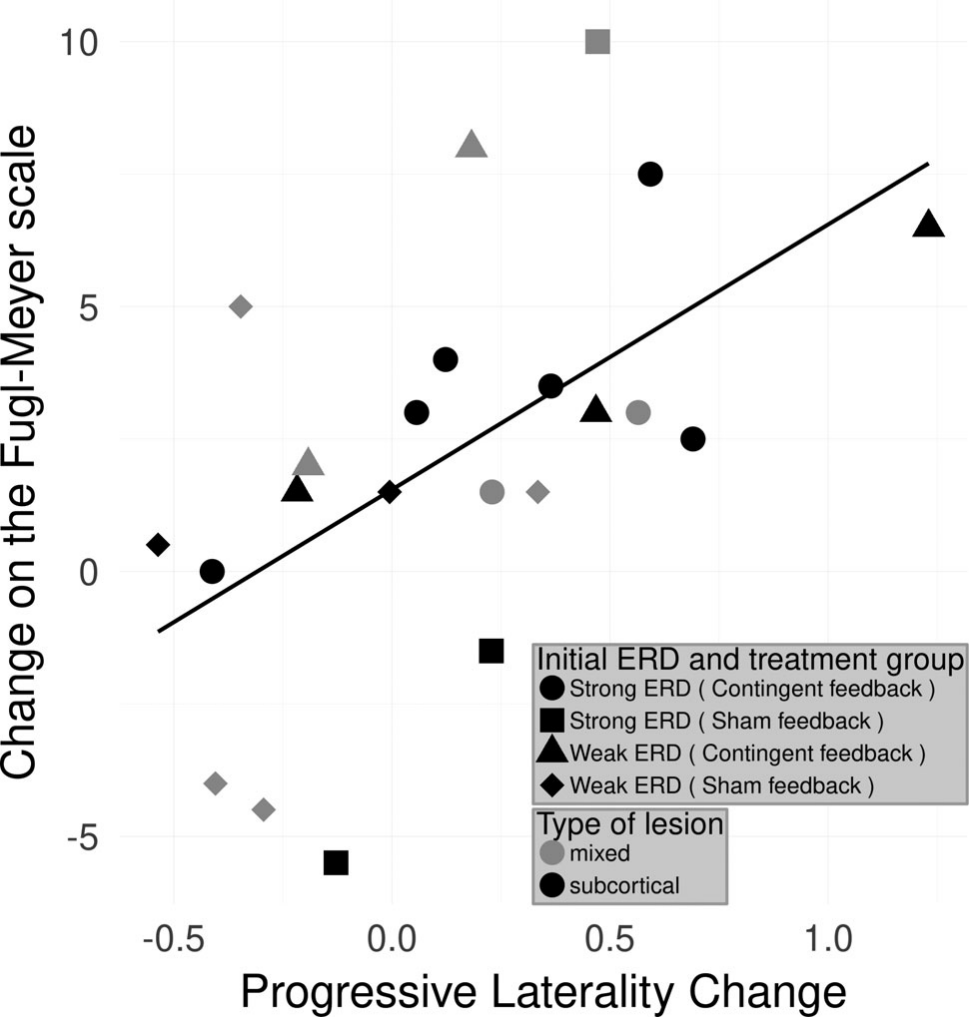
Relationship between improvement and interhemispheric difference of changes of the ERD in the alpha band. Relationship between improvement and interhemispheric difference of changes of the ERD in the alpha‐band: Adjusted *r* ^2^ = 0.279; *F*(1, 20) = 9.11, *p* = .0068. Values on the *x*‐axis express the difference between the progression of the ERD on the healthy hemisphere and the ipsilesional side. Positive values on this axis indicate that throughout the training patients exhibited stronger ipsilesional ERD, negative values indicate a stronger ERD on the healthy hemisphere. The regression indicates that the larger a difference is found the better the motor improvement

To investigate the interhemispheric asymmetry during motor recovery, the progressive laterality coefficient *pLC*_*ERD*_ was used to predict the clinical change ΔcFMA. The *F*‐test for this linear regression equation was significant: *F*(1, 20) = 9.11, *p* = .007 with an adjusted *r*^2^ = .28 (Figure 4). The analysis thus demonstrated that the patients who progressively produce more ipsilesional relative to contralesional brain oscillatory activity (stronger desynchronization) in the alpha band during the course of training improved motor function.

Since the model predicting change on the Fugl‐Meyer scale from the ERD of the contralateral/ipsilesional hemisphere was significant for the individual SMR frequency, we also analyzed the interhemispheric asymmetry in this frequency band. The results and plots are shown in Supporting Information, section 4. In summary, the *F*‐test for this linear regression was not significant but showed a trend (*F*[1,20] = 3.76, *p* = .067). The fit of the model to the data was low: *r* ^2^ = .1161.

## 4 DISCUSSION

We investigated how the brain oscillatory activity of severely impaired chronic stroke patients changes throughout a brain‐controlled robotic intervention for motor rehabilitation of the upper limb and how it relates to the functional motor improvement. We found that dynamics of event‐related desynchronization in the alpha frequency range (ERD) significantly correlate with motor improvement. Most notably, patients showing a relatively strong ERD on the side of the lesion at the beginning of the intervention improved when progressively increasing ERD during movements of the paretic arm in the course of the intervention. Patients showing a relatively weak ERD on the affected hemisphere improved when progressively decreasing ERD. Furthermore, we found larger motor improvements in patients with a progressively larger ERD on the hemisphere of the lesion as compared to concomitant ERD on the healthy hemisphere. The results indicate that the patients might have used two strategies to gain control over the orthoses to link brain oscillatory activity and upper limb movement. Their success rebalancing ipsi−/contralesional activity plays an important role in impairment reduction.

Considering that the proprioceptive feedback was initiated based on the individual SMR ERD, patients who could elicit a strong ERD at the beginning could learn to control the robotic orthosis BMI more easily. We show that a strong ERD on the ipsilesional side during movement attempts of the paretic limb and a subsequent further increase of the ERD was linked to recovery whereas a strong ERD on the healthy hemisphere was not. The results indicate that generating a strong ERD on the hemisphere of the lesion may suffice to regain control of the paretic limb via BMI and to reduce motor impairment. It was indicated that patients without transfer of ERD from the contra‐ to the ipsilesional hemisphere do not improve as predicted by the concept of learned nonuse (Daly & Wolpaw, 2008). A linear relationship between the relative progressions of the ERD on both hemispheres was observed. The modeling presented, links greater improvement of motor function to stronger ERD on the affected hemisphere than on the healthy hemisphere during BMI intervention. Patients with weak ERD during movement attempts of the paretic arm at the beginning of the intervention improved if they showed progressively reduced ERD on the hemisphere of the lesion and an even more pronounced progressive reduction of desynchronization on the healthy hemisphere. One additional explanation to the learned nonuse model of rehabilitation for this phenomenon is that when having acquired proficiency in performing the motor task, reduced ERD represents more efficient inhibition of systems that are not task‐relevant on the ipsilesional side (Klimesch et al., 2007; Taub et al., 1994). Moreover, a connection between alpha synchronization of the EEG and focalized suppression of areas involved in generation of movements irrelevant to the task is assumed (Klimesch et al., 2007; Pfurtscheller, Stancák, & Neuper, 1996). The reduction of desynchronization on the healthy hemisphere as compared to the affected hemisphere indicates less recruitment of the healthy hemisphere during the course of the training as predicted by the model of learned nonuse (Taub et al., 1994). Experiments have shown that interhemispheric inhibition from the healthy to the affected hemisphere is associated with deficient motor recovery (Murase, Duque, Mazzocchio, & Cohen, 2004). Concordant with this interpretation the increased desynchronization of the healthy hemisphere is associated with poorer recovery (Kaiser et al., 2012).

The stratification of the patients into two subgroups (relatively strong and weak ERD) after investigating the linear model paved the way to a concise interpretation of the results obtained. Learning to control a BMI involving proprioceptive feedback modulates desynchronization of the SMR (Ramos‐Murguialday et al., 2012). The group receiving sham feedback might have had a lower or no effect of the practice on the modulation of their SMR. Nevertheless, random correct feedback was sometimes administered, because the orthoses might have also moved while patients correctly produced ERD and both groups received identical physiotherapy after BMI training. Since almost all patients showed some behavioral change, we assumed that the neurophysiological data could explain these changes, which was the main goal of this investigation. The number of patients analyzed did not allow for a robust analysis including stratification by feedback group. There was no difference in number of trials rejected due to EEG or EMG artifacts between the feedback groups. For these reasons we collapsed the analysis across both feedback groups. The oscillatory signature of recovery was our target. A study with a larger number of participants than presented here could potentially uncover whether or not there are differences of the progression of alpha ERD throughout the training depending on if the patients received correct or sham feedback. Behavioral effects of the feedback group have already been investigated in the primary analysis (Ramos‐Murguialday et al., 2013). Moreover, for generalizing it is important to reduce variance in the data caused by noise. Previous work of our group clearly showed the adverse effects of artifacts on the analysis of EEG power where gamma band activity overshadowed activity in the lower bands and suggested ways to avoid or minimize their influence on BMI control and posterior analyses (López‐Larraz et al., 2018). We ensured robustness of the results presented by way of employing the conservative fully automatic rejection procedure.

The longitudinal analysis of the desynchronization of beta oscillation does not allow concise interpretation because the model is not significant. An explanation could be that beta desynchronization is not upheld throughout the whole trial, as has been shown in a healthy population (Ramos‐Murguialday & Birbaumer, 2015). There, significant beta desynchronization only occurred in the beginning of the movement period of the trials. In the present analysis the spectral activity was computed over the whole movement period of trials. Furthermore, we might not be able to capture the dynamics of beta oscillations in terms of linear modeling of desynchronization. More complex metrics such coherence might be more suited (Nicolo et al., 2015). The longitudinal analysis of the individual SMR frequency band shows weaker fits of the model than the analysis of the alpha band. An explanation might be that this analysis included some patients that were rewarded for SMR desynchronization in the beta frequency range during the intervention (see Supporting Information, section 4, Table S2). In healthy populations significant power decreases during execution of a BMI task lasting several seconds have mainly been found in the alpha frequency range (Ramos‐Murguialday & Birbaumer, 2015) and movement‐related activity is known to spread across the whole alpha range (Pfurtscheller & Lopes da Silva, 1999). This result underlines the potential of alpha desynchronization as a biomarker as it explains the variance of the changes in the Fugl‐Meyer scores better than the other frequency ranges.Alpha ERD was also evaluated in the pre and post assessment of the trial. The patients performed repeated movement attempts of their paralyzed arm. We found no difference of alpha ERD between the pre and the post assessment and between groups (see Supporting Information, section 3). The fact that alpha ERD did not change from pre to post despite the behavioral changes could be attributed to the lack of proprioceptive feedback in the assessment and the difference in task (one is controlling the hand open/close movements of an orthosis by way of modulation of their SMR and the other one is a natural attempt to open and close the hand). Since the patients only performed movement attempts and they did not use the exoskeleton that provided them with proprioceptive input (their hands were completely paralyzed) during the pre and post assessments, the sensorimotor activity differs, as this type of feedback influences the modulation of brain rhythms (Ramos‐Murguialday & Birbaumer, 2015). Furthermore, the classifier used in the BMI rewarded differences from the intertrial interval (consider as rest) and the task (attempt to move to downregulate the SMR power and move the orthosis) SMR power using the last 15 s of data for both cases to create the two data distributions (more information can be found in the Supporting Information of the original trial (Ramos‐Murguialday et al., 2013)). Therefore, they could efficiently move the orthosis by either increasing power during inter trial interval, decreasing power during BMI task or both, to decrease variability of the distributions. This fact allowed each patient to choose their own strategy implicitly. These reasons and their initial ERD modulation ability make a general prepost comparison of ERD values complicated without the use of a robotic orthosis. Probably a stratification of this comparison should be done, and unfortunately, the number of patients in the present work limits that comparison and does not allow drawing any conclusion. Due to these results, it might be difficult to generalize or use alpha as biomarker if the screening is not executed with a brain‐controlled orthosis. This finding suggests investigation of alpha ERD in pre‐ and postassessments of movement‐attempts that include proprioceptive feedback in future trials, as passive movements modulate desynchronization (Ramos‐Murguialday & Birbaumer, 2015). Inclusion of passive movements via orthoses could be a complementary measure for assessment of ERD with proprioceptive feedback (as has been already suggested using electrical stimulation [Cho, Vidaurre, Hoffmann, Birbaumer, & Ramos‐Murguialday, 2011]) that would need to be tested in future trials.

Even smallest improvements on the Fugl‐Meyer scale could mean a relevant behavioral change especially in these severely chronically paralyzed patients, in which no spontaneous behavioral improvements are expected. The FMA changes are particularly meaningful for modeling and they are preserved in the long‐term (Ramos‐Murguialday et al., 2019). The test–retest reliability of the Fugl‐Meyer test is very high (Platz et al., 2005), but its sensitivity especially in severe patients might not be sufficient. Therefore, several measures were taken to ensure that the changes in the original study are adequately captured. First, the assessors were blinded to group allocation to avoid a potential retest bias. If there had been a general repetition effect all patients should have improved, which is not the case. Second, the mean of both baseline FMAs was used to measure improvement (Whitall et al., 2010). Statistical analysis of the Fugl‐Meyer values of arm and hand of the two baseline assessments for the present cohort showed that the distributions are not different (Wilcoxon signed‐rank test: *p* = .30). This underlines that the test–retest reliability of the FMA is high in our sample. Third, the assessment focused only on the upper limb motor scores of arm and hand without coordination and speed, and without scores related to reflexes, further reducing variability (Crow & Harmeling‐van der Wel, 2008). Further trials with longer treatment duration or refined methods should boost the behavioral effects to skills of functional relevance. To better understand our results, we repeated our statistical modeling for the arm and hand motor skills separately and observed significant models only for the arm part. This was expected, as most patients had larger motor improvement in the proximal part of the arm. This larger variability in the arm scores is explained by the progression of the ERD in the alpha band, confirming the results obtained with the combined arm and hand Fugl‐Meyer scores. The hand scores alone could not be explained by the linear model, probably because of the lower variability of motor improvement scores (cf., Supporting Information, section 5). In this case, the large impairment of our patients (part of the inclusion criteria) and the low sensitivity and ordinal origin of the Fugl‐Meyer scale limits our modeling. However, trials in acute or low‐to‐mild‐to‐severe patients, and/or longer and refined trials might also increase recovery of hand limb motor skills, which then might also be explainable by ERD progression.

Although cortical integrity is reflected in oscillations of the sensorimotor network measured by ERD, the cortical or subcortical location of the lesion was not a confounder of the modeling procedure (Park, Kwon, Kim, Lee, & Kim, 2016; Ray, Lopez‐Larraz, Figueiredo, Birbaumer, & Ramos‐Murguialday, 2017). First, inclusion of the lesion location as factor did not affect the predictive power of the LMEMs. A likelihood ratio test of a model comparison of a model with and a model without the factor lesion location did not show a significant difference (*χ* ^2^ = 4.25, *p* = .12). Second, LMEMs allow for individual variations of intercept and slope of the progression of the ERD. That is why the relative individual change of ERD throughout the training can be compared between patients with different lesion characteristics. Moreover, in the patients with mixed lesions (subcortical and cortical) damage of the precentral gyrus and the postcentral gyrus did not lead to differences in expression of alpha ERD during the premeasurement (cf., Supporting Information, section 6).

Linear mixed‐effects models are suited for describing physiological data because they acknowledge individual deviations from the population mean and account for unequal number and unequal spacing of data points (Lang et al., 2016). However, each model is a simplification of the data. Learning processes in Neurofeedback have also been described with much higher orders (Gunkelman & Johnstone, 2005). The model coefficients provide the best description of the data in a least squares sense and the LMEM including subject‐specific slopes describes the data significantly better than a model not allowing deviation from the general slope. A likelihood ratio test of a model comparison shows a significant difference (*χ* ^2^ = 6.57, *p* = .038). However, even with the flexibility that linear‐mixed effects models allow, assuming linear progression of the ERD values could be an oversimplified description of the true time course. Moreover, the two‐staged linear modeling employed in the present work could introduce further simplification due to the second modeling step, which might blur the results. On the other hand, linear models allow for the description of the underlying processes with only a few parameters, which is an advantage for intuitive interpretation and quantitative comparison of the models and necessary for the two‐stage analysis employed here.

Four patients of the control group showed a decline of their motor function regardless of the dynamics of their ERD throughout the intervention (two squares below the zero line in Figure 2). It has been suggested that the contingency of brain‐activity and visuo‐proprioceptive feedback is key to cortical reorganization and recovery (Ramos‐Murguialday et al., 2013). Noncontingent feedback interfered with learning and thus could worsen motor impairment (e.g., reinforcing maladaptive synergies), which could happen with open‐loop control of body actuators (e.g., robotics and electromagnetic stimulation) or during physiotherapy.

The present results in severe chronic stroke indicate that EEG oscillatory activity can predict recovery of these patients and links its progression to functional motor recovery, and therefore mark it as a promising biotarget for rehabilitation interventions. In heterogeneous conditions such as stroke, biomarkers could play an important role in informing treatment pathways (informed patients stratification). Mane et al have recently shown that the predictive power of EEG‐based markers may be specific to the intervention methodology (Mane et al., 2019). Studies of oscillatory brain activity during motor imagery and movement of the paretic hand of moderately to severely affected chronic stroke patients (Kaiser et al., 2012) as well as subacute patients of mild to moderate (Platz et al., 2002) and severe impairment (Pichiorri et al., 2015) support our findings suggesting that the level of impairment is negatively correlated to the desynchronization of alpha oscillations on the ipsilesional hemisphere. Moreover, an increase of ipsilesional ERD was observed after spontaneous recovery in acute stroke (Tangwiriyasakul et al., 2014) with concomitant lack of ERD on the healthy hemisphere, which indicates our results might generalize in acute and sub‐acute stroke patients. The sensorimotor ERD magnitude has also been shown to correlate with recovery in spinal cord patients (López‐Larraz, Montesano, Gil‐Agudo, Minguez, & Oliviero, 2015), supporting the validity of this metric as a viable and easily obtainable biomarker of clinical progress in patients suffering from motor impairments and as a measure of brain plasticity (Takemi, Masakado, Liu, & Ushiba, 2015). Moreover, the presence of alpha oscillations at cortical sites of the sensorimotor systems reflects the intact balance of thalamic circuits, particularly reticular thalamic recurrent inhibition of thalamocortical afferents (Steriade, Gloor, Llinas, da Silva, & Mesulam, 1990). Lack of these oscillations in relaxed wakefulness and sleep thus does not allow the excitatory blockade of inhibitory reticular‐thalamic and centro‐thalamic circuits at the ipsilesional thalamo‐cortical system. Reappearance of the delicate excitatory‐inhibitory balance in the thalamocortical circuits after stroke in the course of a learning process directly targeting this oscillatory mechanisms, clearly supports the neurophsyiosological logic of BMI strategies (Birbaumer & Cohen, 2007; Birbaumer, Elbert, Canavan, & Rockstroh, 1990).

Stinear et al. (2017) proved the performance of their sequential algorithm PREP2. It is based on clinical, neurophysiological and neuroimaging markers. Not only does it correctly predict the clinical outcome for 75% of patients after stroke, but it also shows that transcranical magnetic stimulation and clinical tests may replace much more expensive assessments such as magnetic resonance imaging without loss of accuracy. EEG‐based biomarkers of stroke could serve the same purpose of improving treatment outcome while reducing effort. Furthermore, biomarkers of stroke and recovery could also support stratification of participants for clinical trials and thus improve statistical power by reducing unexplainable variance. In the present analysis changes in alpha ERD are only found in the data of the training, which underlines the proprioceptive and longitudinal aspect. Using an orthosis providing proprioceptive feedback when recording data for pre‐ and postassessments could enable an evaluation. This would increase the effort of obtaining the data for the screening but might support patient stratification based on the model presented here: If a patient shows strong ERD in the assessment the intervention could focus on further strengthening of desynchronization. If the patient has less ability to generate ERD on the ipsilesional hemisphere the intervention could focus on bilateral asymmetry. Indeed, all patients might profit from changing the focus of down‐regulating ipsilesional alpha oscillations to modulating the interhemispheric balance of alpha oscillations, which might represent a more beneficial bio‐target (i.e., BMI control signal) for EEG‐based BMI applications in stroke rehabilitation.

Our results constitute a building block of more generalizable statistical models of the process of motor recovery in chronic stroke. However, it is important to emphasize that models as the one presented here only show correlations to outcome variables. Despite the statistical strength of the predictions no causal inference can be made. Therefore, including further neurophysiological markers and clinical information would improve the prediction of outcome, informing procedures and tracking of progress. The present results encourage more efforts to pool data of stroke rehabilitation procedures like the ENIGMA Stroke Recovery initiative (http://enigma.ini.usc.edu/ongoing/enigma-stroke-recovery/) to conceive statistical models that will further improve predictive power of and conclusions drawn from data such as presented here. Quantitative statistical comparison of performance of different markers and different combinations and sequences of markers could eventually yield the optimal procedure and best outcome for the individual patient.

## Supporting information


**Appendix S1**: Supporting Information

## Data Availability

The data that support the findings of this study are available on request from the group's representative, Dr. Ander Ramos‐Murguialday (E‐Mail: ander.ramos‐murguialday@uni‐tuebingen.de). The data are not publicly available due to privacy or ethical restrictions.
