# Supplementary material for: Brain oscillatory activity as a biomarker of motor recovery in chronic stroke
Source: Hum Brain Mapp. 2019 Nov 28;41(5):1296–308. doi: 10.1002/hbm.24876 (PMC7268060; doi:10.1002/hbm.24876)
Supplement: Supplementary file 1 — Appendix S1: Supporting Information [file HBM-41-1296-s001.pdf]

1. Rejection of trials, sessions and subjects

1.1. Extended CONSORT flow diagram

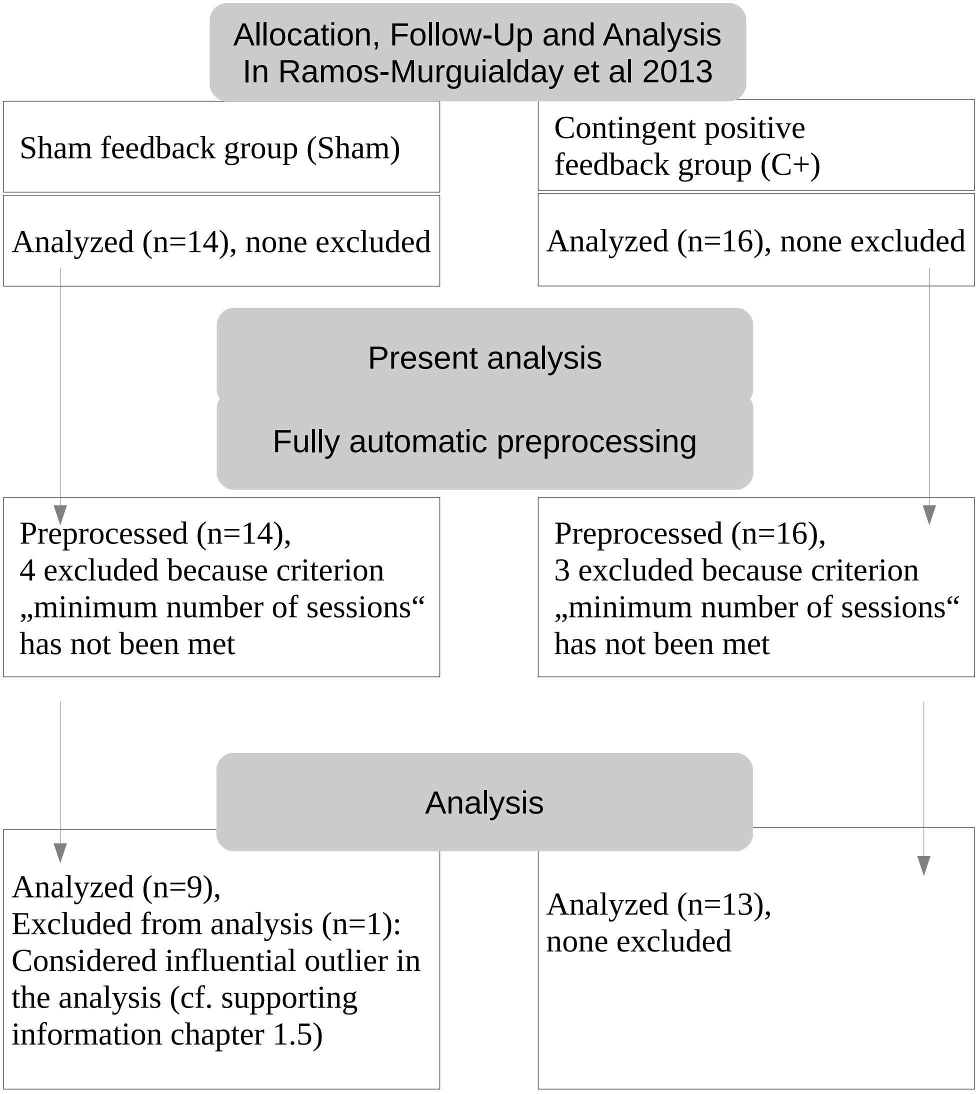

it

Figure S1: Extended CONSORT flow diagram
All patients of the final pool of patients of the main study (Ramos-Murguialday et al. 2013) were included in the present analysis with the same allocation to feedback groups. After the rejection procedure in the automatic preprocessing step four patients were removed from the "Sham" group and three patients were removed from the "C+" group. Moreover, one patient was considered an influential outlier and excluded from the analysis.

1.2. Rejection details

Table S1. Rejection details

Subject ID	Total # sessions	# sessions removed	Total # trials	# trials removed	Feedback group	Was subject part of the analysis?
**1**	16	4	2731	2296	C+	yes
**2**	17	10	2828	2617	Sham	no
**3**	19	5	3188	2619	Sham	yes
**4**	16	2	2680	1913	Sham	yes
**5**	16	7	2570	2102	C+	yes
**6**	19	18	2978	2908	Sham	no
**7**	17	6	3401	2845	C+	yes
**8**	16	8	2553	1870	C+	yes
**9**	17	1	3430	2139	C+	yes
**10**	19	3	3158	2466	C+	yes
**11**	16	2	2766	2146	C+	yes
**12**	18	9	2920	2544	Sham	yes
**13**	17	2	3003	1948	Sham	yes
**14**	17	11	2889	2688	C+	no
**15**	17	14	2405	2252	C+	no
**16**	11	1	1780	1524	Sham	yes
**17**	12	7	1926	1800	Sham	no
**18**	17	16	2540	2419	C+	no
**19**	18	4	2776	2307	C+	yes
**20**	18	0	2998	1856	C+	yes
**21**	16	5	2555	1554	C+	yes
**22**	19	4	3184	1910	Sham	yes
**23**	15	6	2500	2225	C+	yes
**24**	19	15	2820	2628	Sham	no
**25**	16	5	2624	2219	Sham	yes
**26**	17	3	2666	2097	C+	yes
**27**	18	0	2700	2018	Sham	yes
**28**	17	1	2902	1813	Sham	yes
**29**	18	5	3236	2848	C+	yes
**30**	18	7	2850	1777	Sham	yes

Total number of subjects included in the analysis: 23
Total number of subject included by feedback group: C+: 13 / Sham: 10

The table shows the total number training sessions and the total number of movement attempt trials carried out in the whole training per patient. Moreover, the number of trials and the number of sessions that have been removed from the analysis during the preprocessing are presented. The rightmost two columns indicate the feedback group the patients belonged to and whether or not they were included in the analysis.

Figure S2. Rejection of trials per session in the experimental group

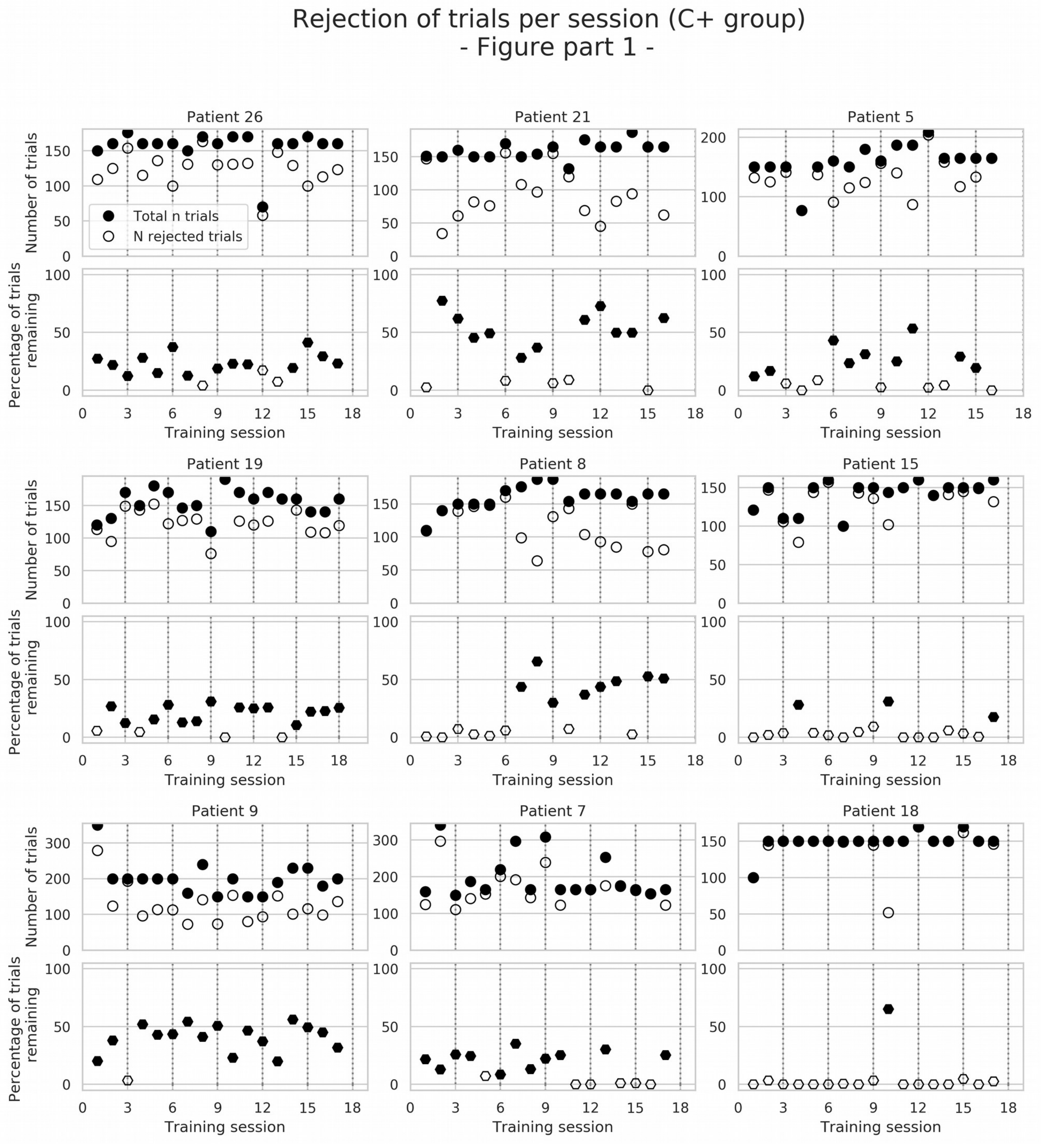

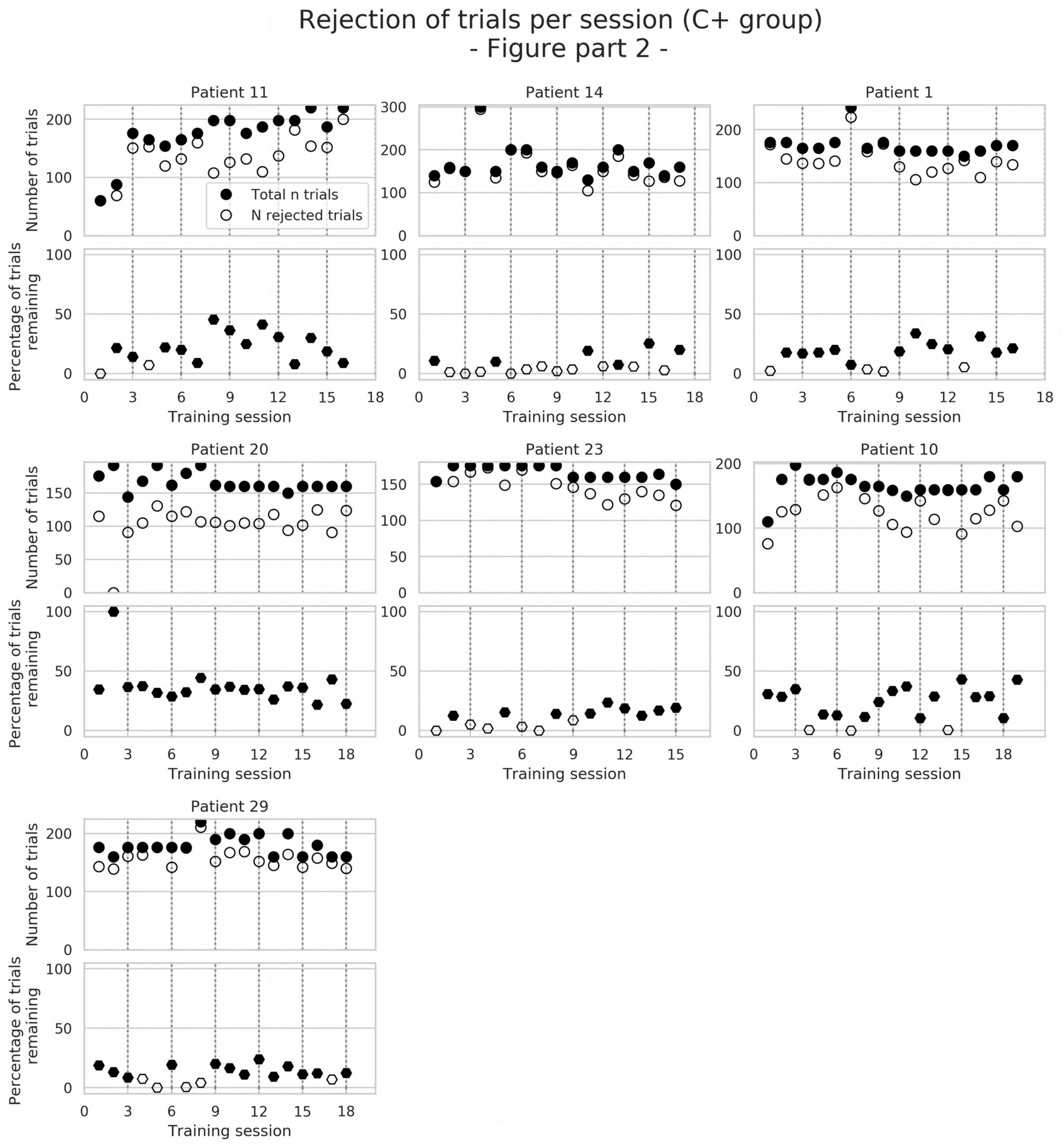

The figure shows the number of trials that have been removed in each training session for each patient (Contingent positive feedback group). The subplots on the top show the total number of trials (full circles) and the number of trials rejected (empty circles) per session. The bottom subplots show the percentage of trials remaining after the rejection procedure. The globally defined minimum number of trials necessary for a session to remain in the analysis is 15. The sessions having less than 15 'good' trials remaining have been rejected. Rejection is indicated by empty hexagons. The percentage threshold for trial rejection varies between sessions because of the total number of trials varies per session.

Figure S3. Rejection of trials per session in the sham group

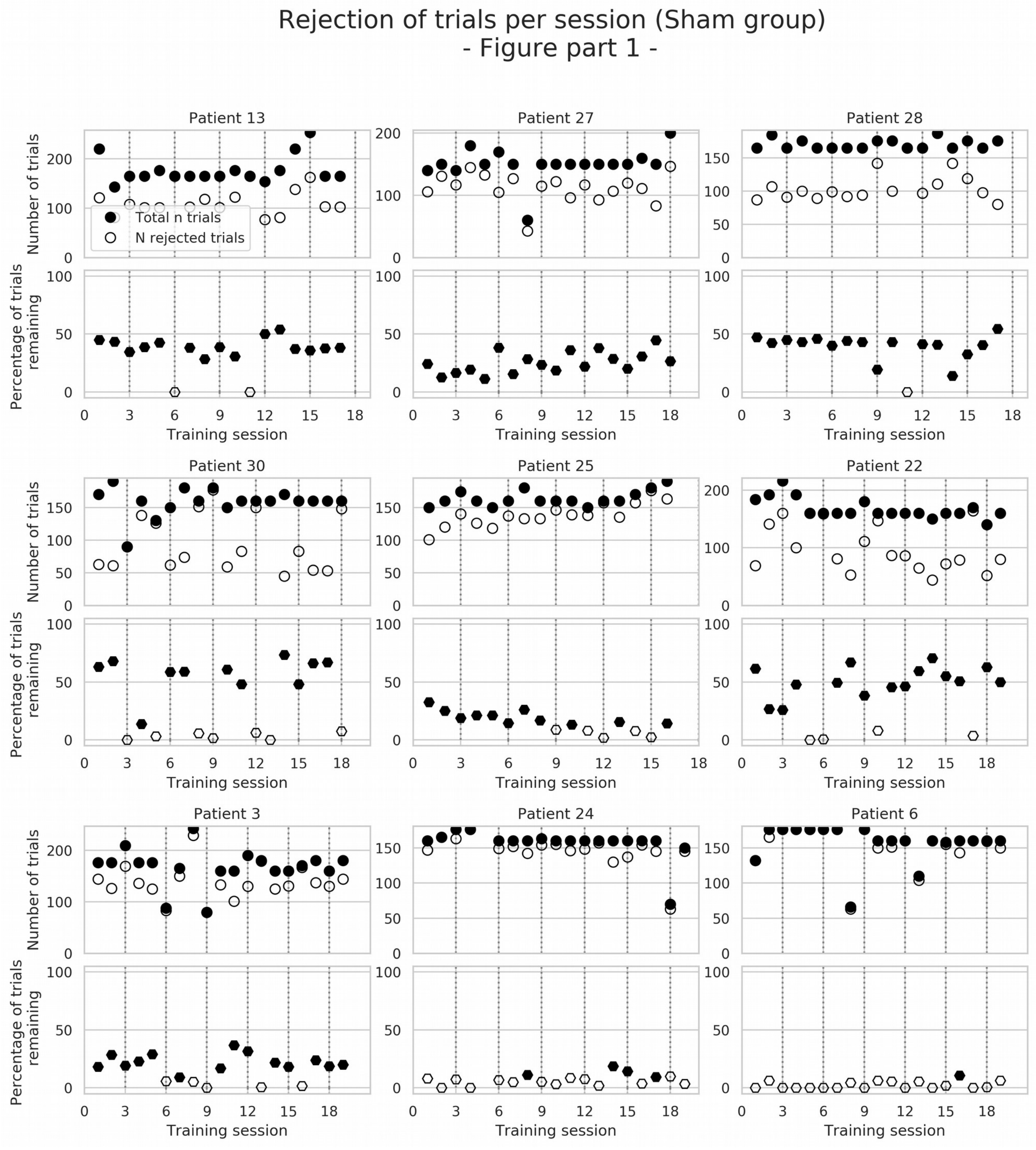

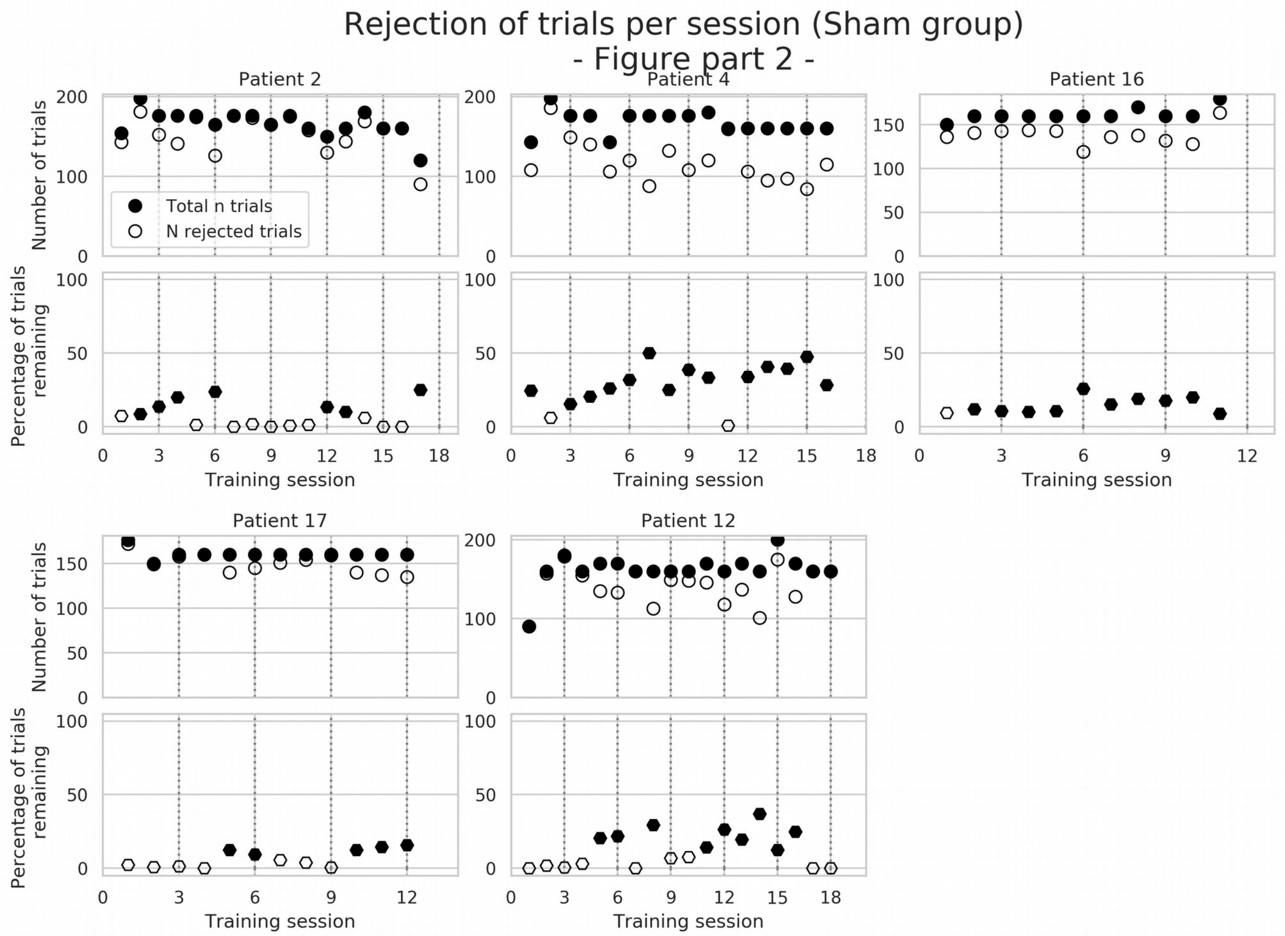

The figure shows the number of trials that have been removed in each training session for each patient (Sham feedback group). The subplots on the top show the total number of trials (full circles) and the number of trials rejected (empty circles) per session. The bottom subplots show the percentage of trials remaining after the rejection procedure. The globally defined minimum number of trials necessary for a session to remain in the analysis is 15. The sessions having less than 15 'good' trials remaining have been rejected. Rejection is indicated by empty hexagons. The percentage threshold for trial rejection varies between sessions because of the total number of trials varies per session.

1.3. Descriptive statistics on rejection

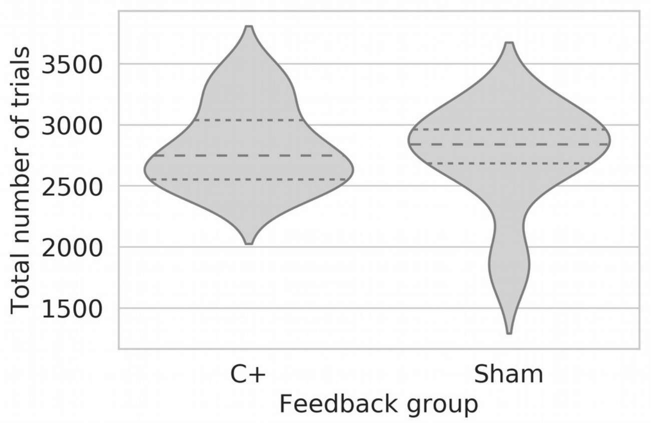

Figure S4. Distribution of the total number of trials
The violin plot displays median and first and third quartile of the data and a density estimation of the distributions of the data. There is no difference between the groups in the total number of trials. There are two Sham patients who had only 12 sessions in total which explains the bottom tail of the plot on the right.

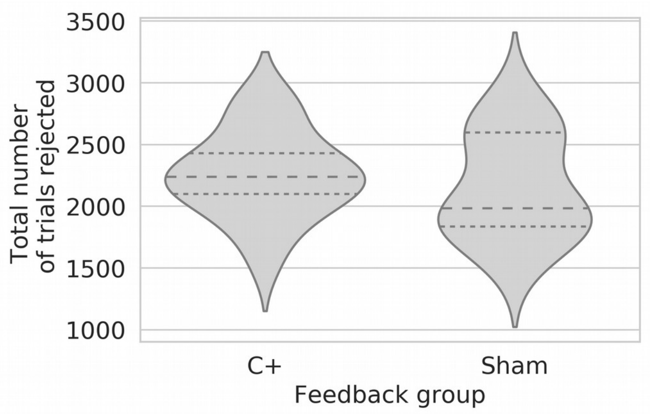

Figure S5. Distribution of the total number of rejected trials
The violin plot displays median and first and third quartile of the data and a density estimation of the distributions of the number of rejected trials of both groups.

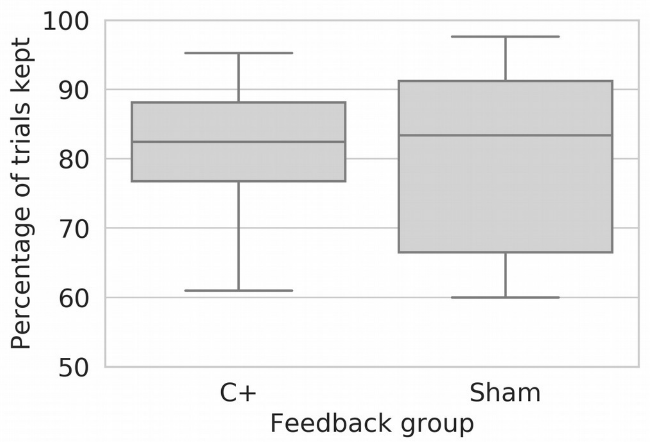

Figure S6. Distribution of percentage of trials remaining per session
The box plot indicates that there is no difference in the percentage of trials remaining per session after preprocessing between the feedback groups. This is confirmed by the result of a t-test: t=0.19, p=0.84.

1.4. Descriptive statistics on reasons for rejection

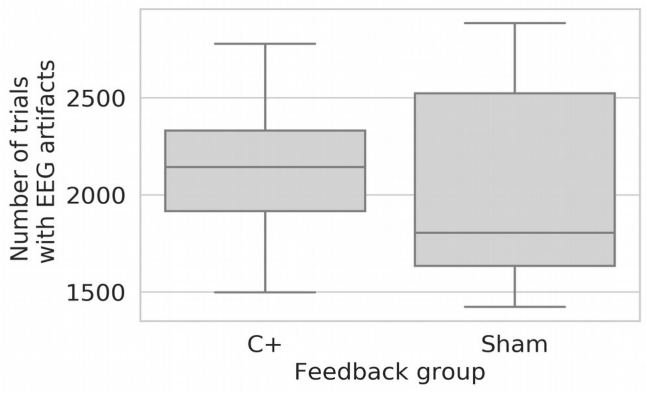

Figure S7. Distribution of the total number of trials showing EEG artifacts
The box plot shows the distribution of the total number of trials per subject with artifacts in the EEG. An t-test does not show a difference between groups: t=0.79, pvalue=0.44.

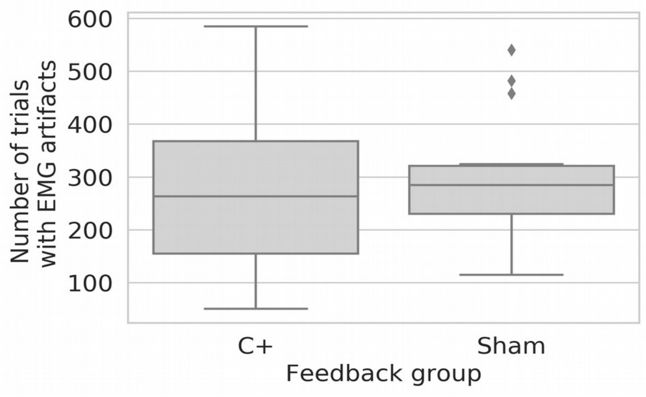

Figure S8. Distribution of the total number of trials showing ipsilateral EMG artifacts
The box plot shows the distribution of the total number of trials removed per subject with artifacts caused by movements of the unaffected arm during movement attempts of the paralyzed arm. There is no difference between groups.

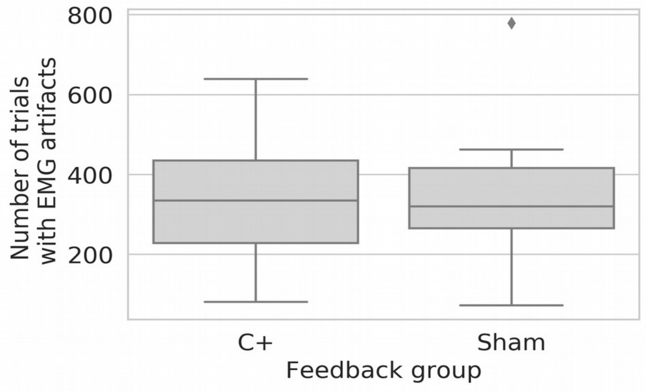

Figure S9. Distribution of the total number of trials showing ipsilateral or bilateral EMG artifacts
The box plot shows the distribution of the total number of trials per subject with artifacts caused by movements of any arm during the rest period. There is no difference between groups.

1.5. Rejection of subject 12

To further improve interpretability and generalizability the R function *dfbetas* was used to investigate the linear models for influential data. The function computes the standardized difference of parameter estimates between a regression model based on the full data and a model from which a subset of potentially influential data is removed.

Dfbetas is defined as:

$$DFBETAS_{pZ} = \frac{b_Z - b_{-pZ}}{se(b_{-pZ})}$$

where the denomiator is the difference between the slope estimate of one of the predictors (Z). The first term (b_z) is the estimate using the full sample and the second term (b_{-pZ}) is the estimate after excluding a patient (p). The value is normalized by dividing the standard error of the second term (Van der Meer et al., 2010).

Here, one out of all subject is left out in each iteration. A DFBETAS value is computed for each parameter in the model. For the linear model the parameter “slope” of the data of the α-band was strongly influenced by subject 12. Removing the subject would change the slope coefficient of the model almost one order of magnitude more than removing the next most influential data point. Moreover, the inspection of the mean power values of the EEG revealed that this patient had, on average, larger power during the movement phase than during the resting period in all training sessions except one (Figure S10). This might indicate that the patient consistently synchronized the SMR during movement attempts or produced SMR synchronization due to an unfiltered artifact (e.g. subtle contractions of the neck or face muscles). On top of that, the patient was part of the control group, which received sham feedback. In the first three sessions, this patient received contingent negative feedback because of a technical issue. In this type of feedback the robot moved whenever the patients did not desynchronize or synchronized their ipsilesional SMR. Synchronization thus could have been rewarded in this patient in the beginning of the intervention. We thus conclude that the patient was not able to and did not learn to produce the desired SMR desynchronization throughout the course of the intervention, which would explain the odd coefficients of the linear mixed model.

We considered the patient an outlier and decided to remove this subject from the analysis.

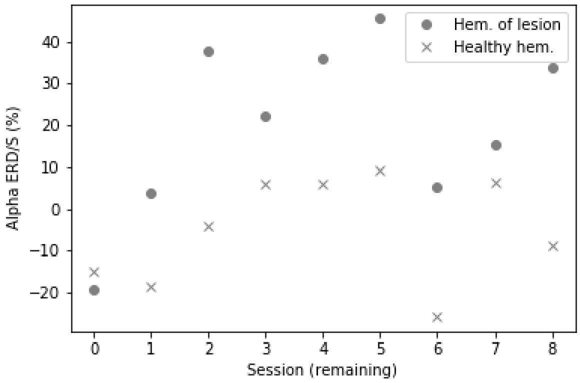

Figure S10. ERD/S values of subject 12
Averaged event-related desynchronization / synchronization in the sessions remaining after the preprocessing (re-indexed from 0 to 8) on both hemispheres. The subject only desynchronized in one of the nine sessions.

2. Modeling procedure

Figure S11 provides a visual description of the two-stage linear modeling procedure (Figure S11).

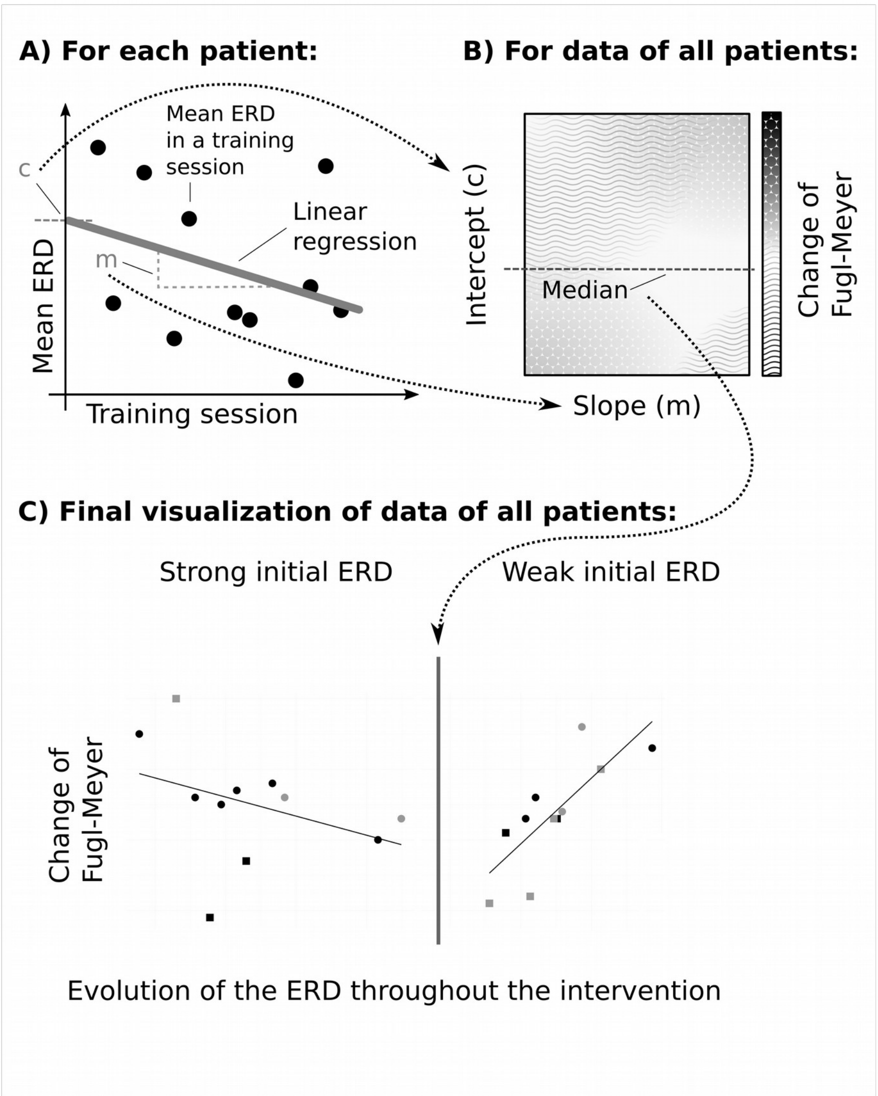

Figure S11. Visual description of the two-stage modeling procedure

The two-step modeling approach for describing the relationship between brain activity (session-wise mean ERD) and the clinical outcome (cFMA).

Step (A): Longitudinal modeling and coefficient extraction. First, the coefficients “intercept” and “slope” are extracted from a linear mixed-effects model for each patient. The linear mixed model provides the best (least squares) estimates of these coefficients. As the initial ERD values and their progression vary for each patient, the factors *time* and *subject* are considered as random effects in the model. Therefore, the two coefficients represent the patient-specific estimated initial value and the progression of the ERD throughout the intervention.

Step (B): Two-dimensional visualization of the interaction in the model. The extracted coefficients of (A) are used in a linear model predicting the clinical outcome (cFMA). The model includes an interaction term. The model thus can capture modulation of the outcome variable by both independent variables. One of the variables might predict the outcome variable depending on the other independent variable. Here, for example, large Fugl-Meyer values are predicted by large slopes if the intercept is large, too.

Step (C): Visualization for interpretation. The median of the intercept values is used to form cross-sections of the data that are visualized in separate panels to increase interpretability of the linear model.

3. Pre and post comparisons of primary outcome measures with new cohort

In this section pre and post comparisons of the primary outcome measures are shown for completeness. Only the 22 subjects used in the main analysis were considered.
Figure S12 shows the comparison of mean event-related desynchronization (ERD) during the pre measurement and the post measurement. At these two time points subjects were seated and they performed cued opening attempts of the paretic hand and resting in randomized order. Data acquisition and processing was the same as for the main analysis. However, there was no minimum number of trials remaining after the analysis and no subject was removed. In an additional preprocessing step ERD values below -100 and above 100 were considered artifacts and were discarded (mean±sd 2.6 ± 2.6 trials per subject contained such values).
Figure S13 shows the comparison of the difference of the modified Fugl-Meyer assessment scores from pre to post stratified by feedback group. A t-test reveals a trend towards a difference between groups (t=2.05, p=0.057).

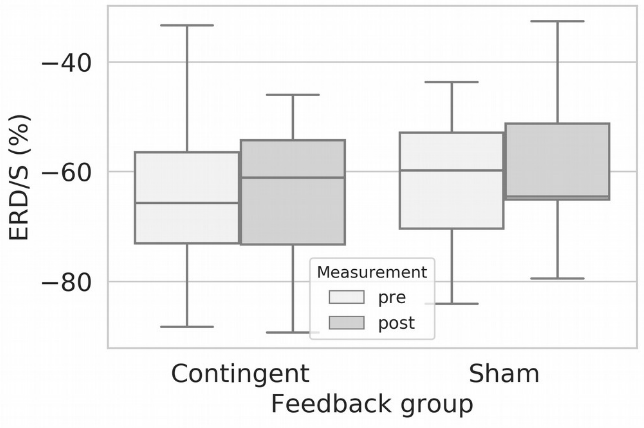

Figure S12
Comparison of alpha ERD values of patients in the two feedback groups during pre and post assessments

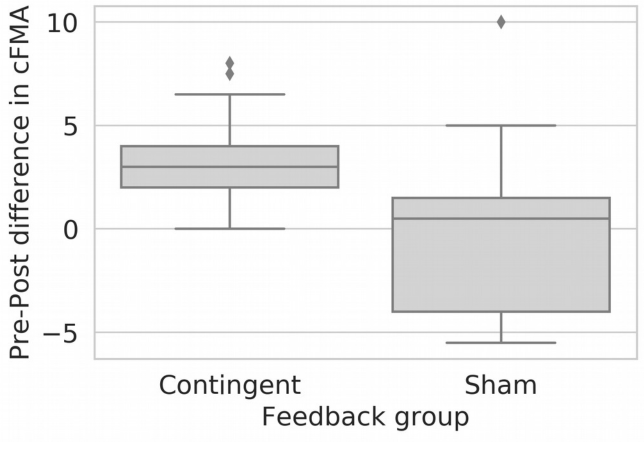

Figure S13
Comparison of the difference of the modified Fugl-Meyer assessment scores from pre to post by feedback group

4. Progression of desynchronization in other frequency ranges than alpha

4.1. Beta frequency range

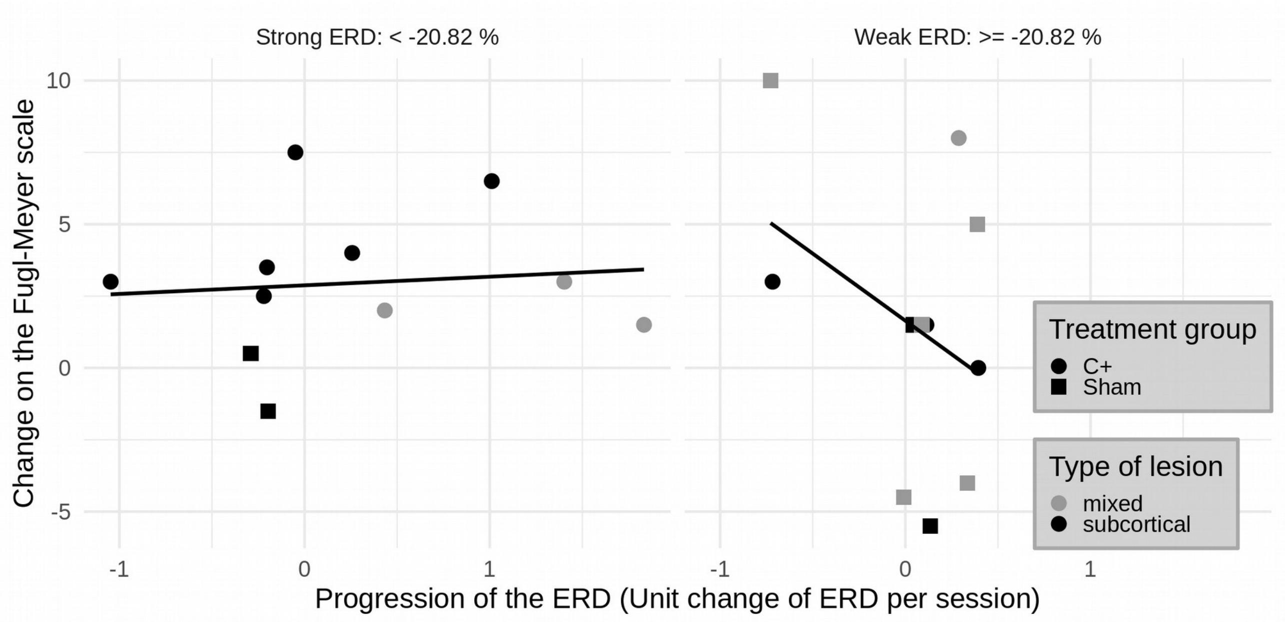

Figure S14: Linear model predicting the improvement of motor function (ΔcFMA) on the hemisphere of the lesion
Linear model predicting the improvement of motor function (ΔcFMA) by the initial ERD and the progression of the ERD of the beta frequency range on the ipsilesional hemisphere over sessions. For improved visualization of the effects of both explanatory variables in the model the patients are separated into two cross-sections showing relatively strong initial ERD (left panel) and a second group showing relatively weak initial ERD (panel on the right). The F-test of the regression equation was not significant.

The linear model for the ERD in the beta frequency range (12 - 25 Hz) was constructed in the same way as the model of the ERD in the alpha frequency range presented in the main document. ΔcFMA is predicted by the coefficients extracted from the corresponding linear mixed effects model: the progression of the ERD throughout the intervention sessions and the initial ERD magnitude. An interaction term was included in the LM to investigate if the initial ERD correlated with the progression of the oscillatory activity. The F-test of the regression equation was not significant and the fit of the model was very low: F(3, 18) = 1.23, p = 0.3278 and an adjusted r^2 = 0.032.

4.2. Individual SMR frequency range

The linear model for the ERD in the individual SMR frequency range was constructed in the same way as the model of the ERD in the alpha frequency range presented in the main document. ΔcFMA is predicted by the coefficients extracted from the corresponding linear mixed effects model: the progression of the ERD throughout the intervention sessions and the initial ERD magnitude. An interaction term was included in the LM to investigate if the initial ERD correlated with the progression of the oscillatory activity (Figure S15). Table S2 shows the frequency range

selected for each subject for control of the exoskeleton. The F-test of the regression equation was significant: $F(3, 18) = 3.475$, $p = 0.038$ and an adjusted $r^2 = 0.26$.

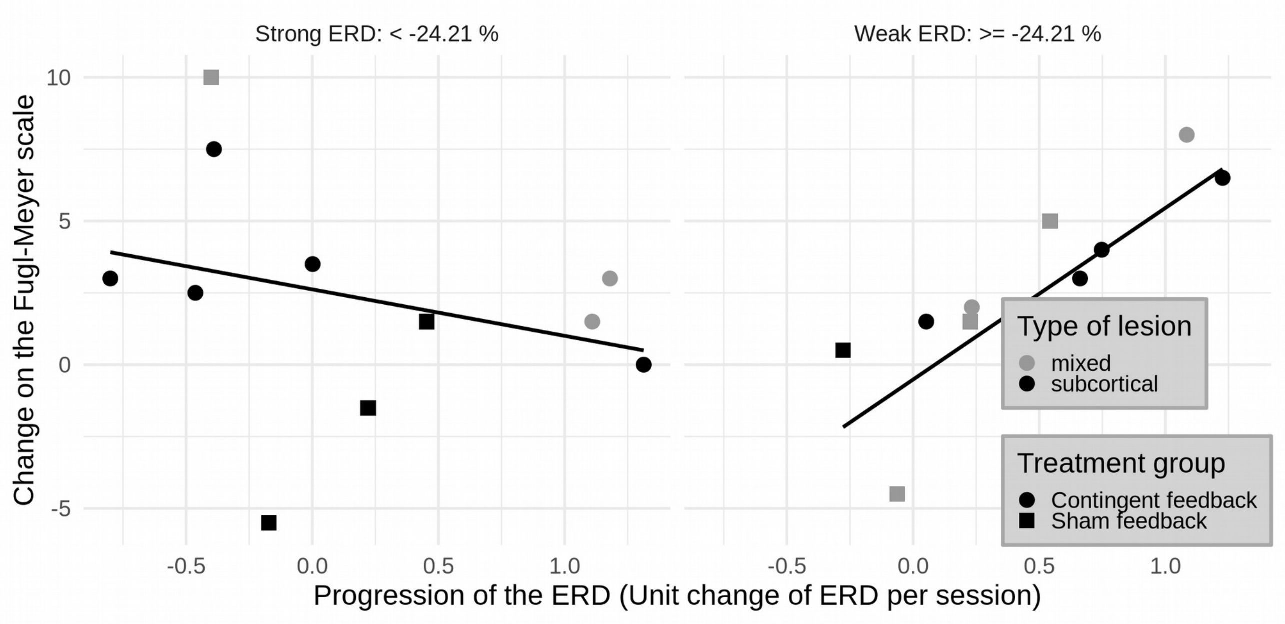

Figure S15: Linear model predicting the improvement of motor function (ΔcFMA) on the hemisphere of the lesion

Linear model predicting the improvement of motor function (ΔcFMA) by the initial ERD and the progression of the ERD of the individual SMR frequency range on the ipsilesional hemisphere over sessions. For improved visualization of the effects of both explanatory variables in the model the patients are separated into two cross-sections showing relatively strong initial ERD (left panel) and a second group showing relatively weak initial ERD (panel on the right).

Table S2: Frequency ranges of the individual SMR

**Subject ID**	**Frequency range of the individual SMR**	**Subject ID**	**Frequency range of the individual SMR**
1	5.5 - 8.5 Hz	19	8.5 - 11.5 Hz
3	8.5 - 11.5 Hz	20	5.5 - 8.5 Hz
4	14.5 - 17.5 Hz	21	8.5 - 11.5 Hz
5	8.5 - 11.5 Hz	22	8.5 - 11.5 Hz
7	11.5 - 14.5 Hz	23	17.5 - 20.5 Hz
8	14.5 - 17.5 Hz	25	8.5 - 11.5 Hz
9	5.5 - 8.5 Hz	26	8.5 - 11.5 Hz
10	5.5 - 8.5 Hz	27	5.5 - 8.5 Hz
11	8.5 - 11.5 Hz	28	8.5 - 11.5 Hz
13	23.5 - 26.5 Hz	29	5.5 - 8.5 Hz
16	8.5 - 11.5 Hz	30	8.5 - 11.5 Hz
		**Average:**	**10.6 +/- 4.8 Hz**

The table shows the frequency range chosen as individual SMR frequency band for control of the exoskeleton in the original study.

The analysis of the interhemispheric asymmetry in the SMR frequency investigated if the progressive laterality coefficient predicts the clinical change ΔcFMA (Figure S16). The F-test for this linear regression equation was not significant but revealed a trend. However, the fit of the equation is low. $F(1,20) = 3.76$, $p = 0.067$ and an adjusted $r^2 = 0.1161$.

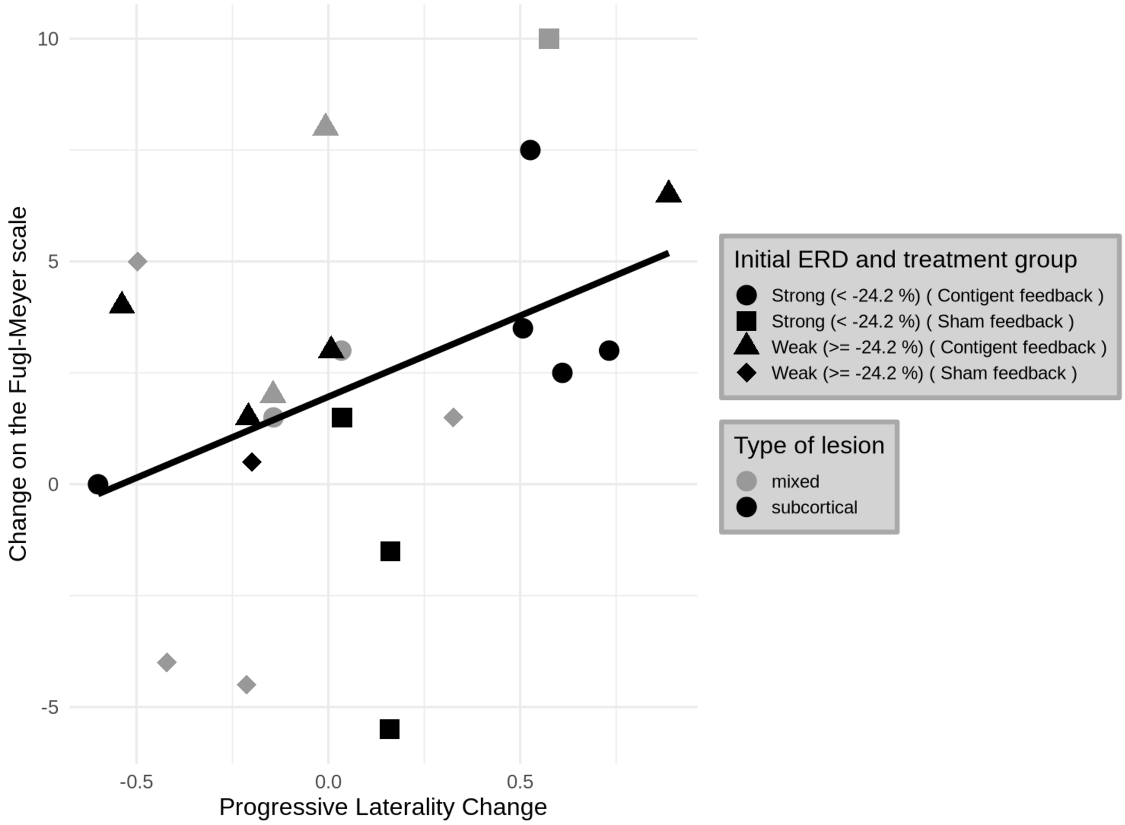

Figure S16: Relationship between improvement and interhemispheric difference of changes of the ERD in the alpha band

Relationship between improvement and interhemispheric difference of changes of the ERD in the individual SMR-band. Values on the x-axis express the difference between the progression of the ERD on the healthy hemisphere and the ipsilesional side. Positive values on this axis indicate that throughout the training patients exhibited stronger ipsilesional ERD, negative values indicate a stronger ERD on the healthy hemisphere. The regression indicates that the larger a difference is found the better the motor improvement.

5. Progression of desynchronization of alpha oscillations with breakdown of FMA scores

5.1. Breakdown of the Fugl-Meyer assessment

The Fugl-Meyer Assessment evaluates impairments in sensorimotor function. Here, the scale was modified to exclude coordination, reflexes and speed. These measures introduce variability since the patients could not actively extend their fingers (Ramos-Murgialday et al., 2013).

The evaluation took into account the motor skills of upper arm and forearm. 15 items were tested with a maximum score of 30 points. Furthermore, the motor skills of hand and fingers were tested comprising 12 items with a maximum score of 24 points. The total maximum score was 54 points.

5.1.1. The movements of the arm test

The following 18 movements were assessed:

Movements 1 ot 6: Synergies of the flexors („touch the ipsilateral ear“):

- elevation
- shoulder retraction, abduction, external rotation
- forearm supination

Movements 7 to 9: Synergies of the extensors (”touch the contralateral knee“):

- shoulder adduction, internal rotation
- elbow extension
- forearm pronation

Movement 10: „Move the hand to lumbar spine“

Movement 11: Shoulder flexion 0-90°

Movement 12. Pro/supination while elbow is in flexion

Movement 13: Shoulder abduction 0-90°

Movement 14: Shoulder flexion 90-180°

Movement 15: Pro/supination while elbow is in extension

5.1.2. The movements of the hand test

The following 12 movements were assesed:

Movement 1: Stabilitby of the wrist in 15° extension while elbow is at 90°

Movement 2: Stability of the wrist in flexion and extension while elbow is at 90°

Movement 3: Stability of the wrist in 15° extension while elbow is at 0°

Movement 4: Flexion/extension of the wrist while elbow is at 0°

Movement 5: Circumduction of the wrist

Movement 6: Flexion of the fingers

Movement 7: Extension of the fingers

Movement 8: Grasping against resistance with metacarpophalangeal joints of digit two and flexion of the proximal interphalangeal joints

Movement 9: Grasping of a scrap of paper

Movement 10: Grasping of a pencil

Movement 11: Grasping of a cylinder

Movement 12: Grasping of a tennis ball

5.1.3. Improvement in arm and hand

Figure S17 shows that the range of improved points on the arm subscore of the Fugl-Meyer assessment is greater than that of the hand subscore (Figure S17).

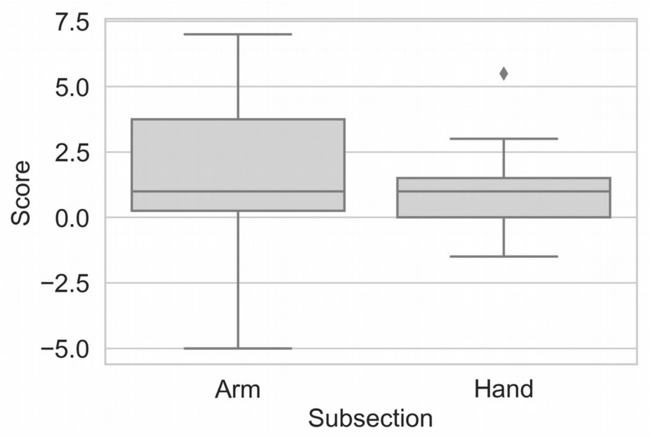

Figure S17: Changes of the scores of the FMA from pre to post measurements separated into arm and hand subsections

5.2. Statistical modeling

The statistical modeling analysis described in the main paper was repeated with a breakdown of the Fugl-Meyer assessment into arm subscores and hand subscores.

The linear models for the ERD in the alpha frequency predicted the change in the arm scores of the Fugl-Meyer assessment (ΔFMA_arm, figure S18) and the hand scores (ΔFMA_hand, figure S19). For the arm scores the F-test of the regression equation was significant: F(3, 18) = 5.15, p = 0.0096 and an adjusted r^2 = 0.37. The F-test of the regression equation of the hand scores was not significant: F(3, 18) = 0.59, p = 0.6293 and an adjusted r^2 = -0.06.

Linear models predicting both FMA subscores from the contralesional hemisphere were constructed. Only the F-test for the regression of the arm subscores for those subjects with relatively weak initial ERD was significant: F(1,9) = 6.4, p = 0.032 and an adjusted r^2 = 0.35 (Figure S20).

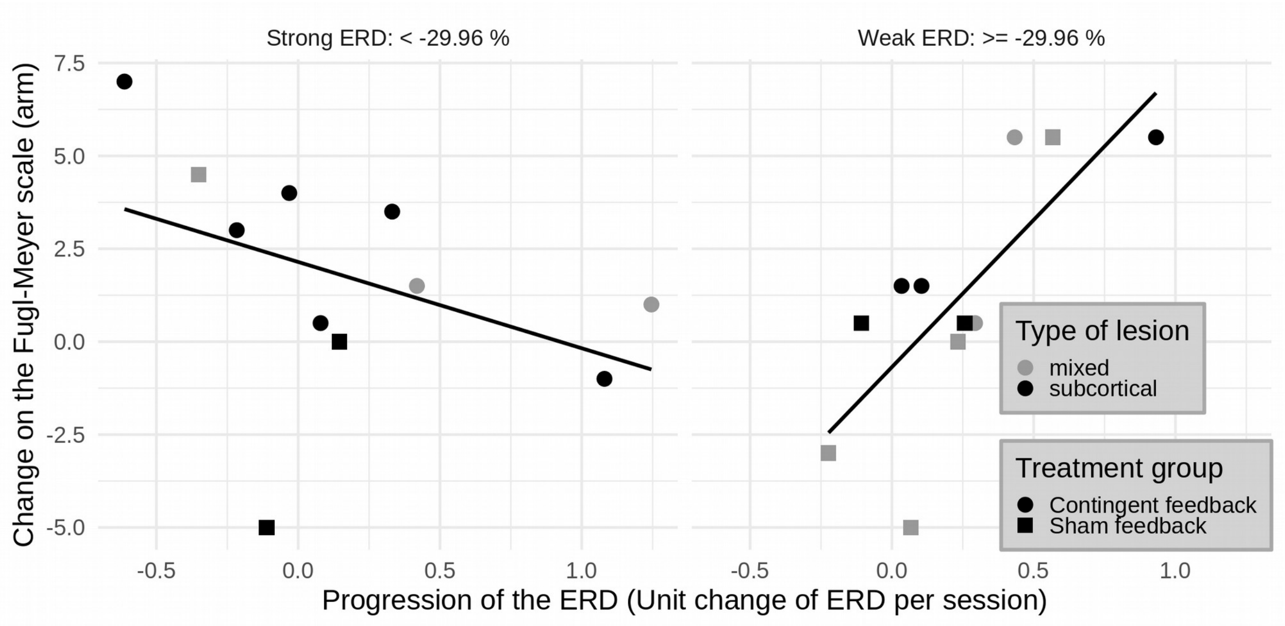

Figure S18: Linear model predicting the improvement of motor function (ΔFMA_arm) on the hemisphere of the lesion

Linear model predicting the improvement of motor function (ΔFMA_arm) by the initial ERD and the progression of the ERD of the alpha frequency range on the ipsilesional hemisphere over sessions. For improved visualization of the effects of both explanatory variables in the model the patients are separated into two cross-sections showing relatively strong ERD (left panel) and a second group showing relatively weak initial ERD (panel on the right).

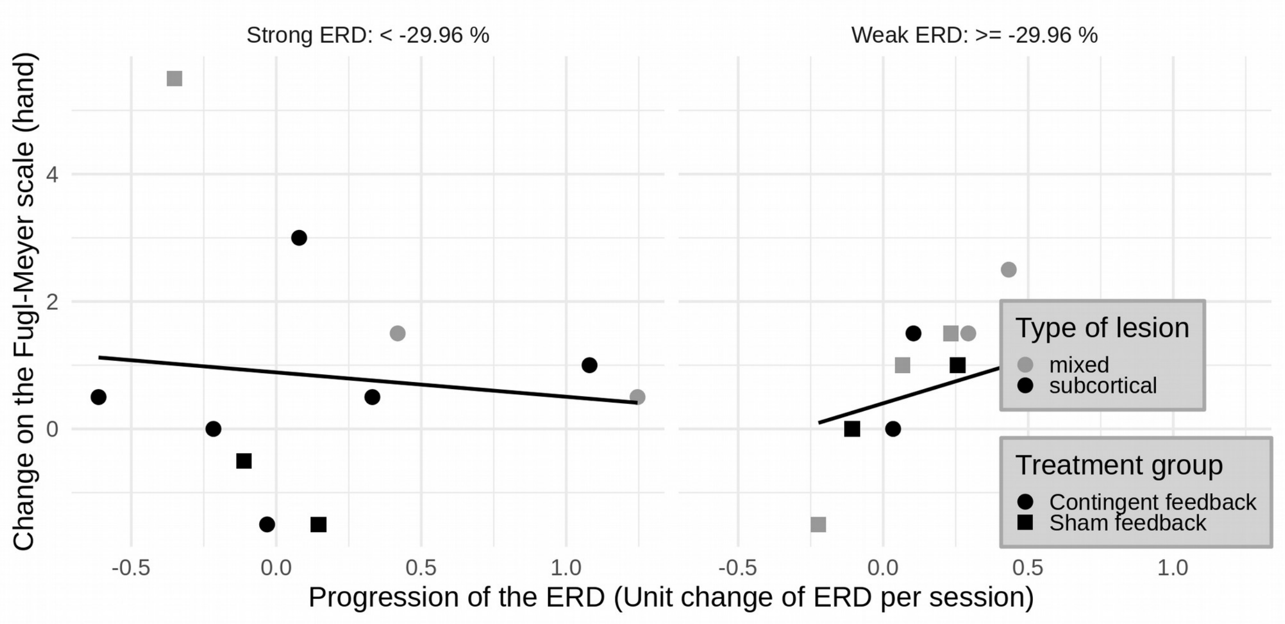

Figure S19: Linear model predicting the improvement of motor function (ΔFMA_hand) on the hemisphere of the lesion

Linear model predicting the improvement of motor function (ΔFMA_hand) by the initial ERD and the progression of the ERD of the alpha frequency range on the ipsilesional hemisphere over sessions. For improved visualization of the effects of both explanatory variables in the model the patients are separated into two cross-sections showing relatively strong ERD (left panel) and a second group showing relatively weak initial ERD (panel on the right).

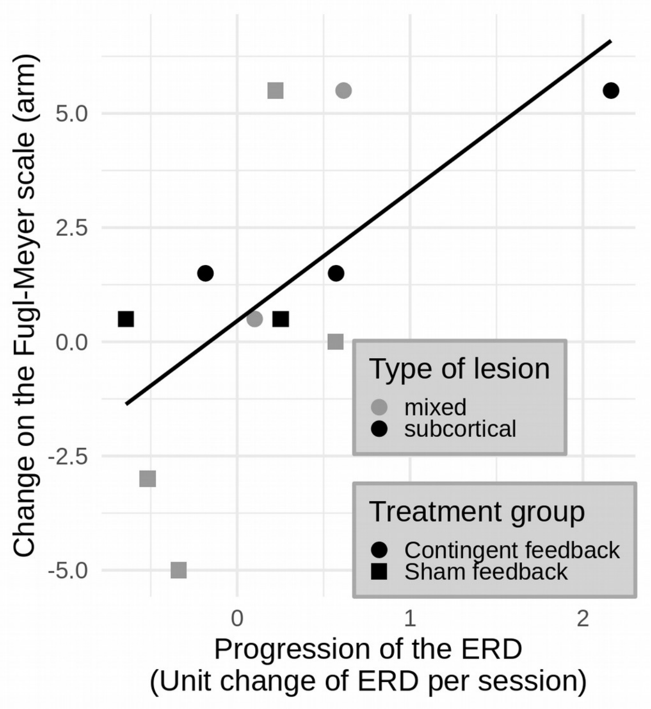

Figure S20: Linear model predicting the improvement of motor function (ΔFMA_arm) on the healthy hemisphere

Linear model predicting the improvement of motor function (ΔFMA_arm) by the progression of the ERD of the alpha frequency range on the healthy hemisphere over all sessions for the patients showing relatively weak initial ERD on the ipsilesional hemisphere. Better recovery was achieved when the ERD on the healthy hemisphere decreased in the course of the training.

6. Lesion characteristics: Involvement of the pre/postcentral gyrus

The precentral and the postcentral gyrus are the locations of the primary motor cortex and the primary somatosensory cortex. Previous works showed that cortical integrity is reflected in oscillations of the sensorimotor network measured by ERD if stroke patients with damaged cortex are compared to patients with subcortical lesions (Park et al., 2016; Ray et al., 2017). This section provides information on the numbers of subjects with lesions reaching the cortex that had the central gyri involved and how this might have affected the relative EEG measured.

Of those subjects who had cortical involvement of the stroke there were eight with and one without involvement of the precentral gyrus. The same numbers apply to the subjects regarding the postcentral gyrus. There were seven subjects who had both gyri affected and only two who did not (Figure S21).

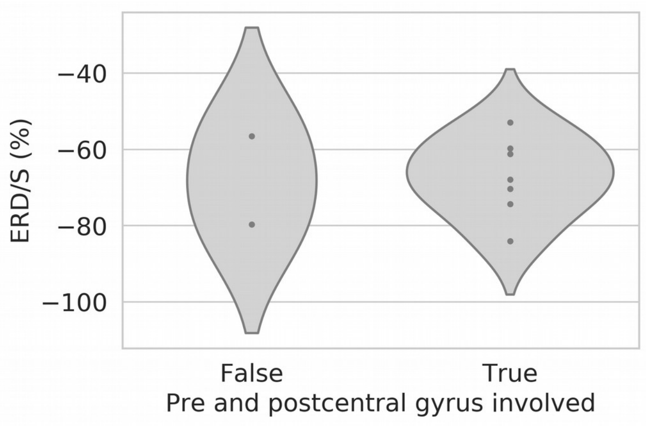

Figure S20: Difference in alpha-ERD over the lesion

There is no difference of the alpha-ERD over the lesion between those subjects with cortical involvement in which the pre/postcentral gyrus is affected by the lesion and those in which it is not. Please not, however, that there are only two subjects in the left distribution.

7. Time-frequency visualization

In this section time-frequency plots of two patients of the subgroup with relatively strong and two patients of the subgroup with relatively weak initial desynchronization are shown. The chosen subjects represent the extremes of the modeled ERD progression (Figure S22). The plots show the time-frequency representation of an early and a late session (Figure S23). The sessions that are plotted here reflect the modeled progression of the ERD. Plots of other sessions might not reflect the modeled progression because the ERD values vary around the slope of the model. The plots were created using Morlet wavelets for the time-frequency decomposition with a resolution of 0.5 Hz from 2 Hz to 36 Hz. The data of the instruction phase of the trials were disregarded and is shown here in light grey. Desynchronization / synchronization was computed as explained in the main document, section 2.3. It is important to note that these values were obtained using Welch's method as described in section 2.3 rather than the time-frequency decomposition by Morlet wavelets used here for plotting. Furthermore, the ERD/S values were computed using the full last four seconds of the rest period as baseline. Here, the baseline period was truncated by 250 ms at the beginning and at the end to alleviate potential aliasing effects of the wavelets. This is the reason why the ERD/S values printed onto the plots may not be exactly the same as the mean of the values within the rectangle shown.s

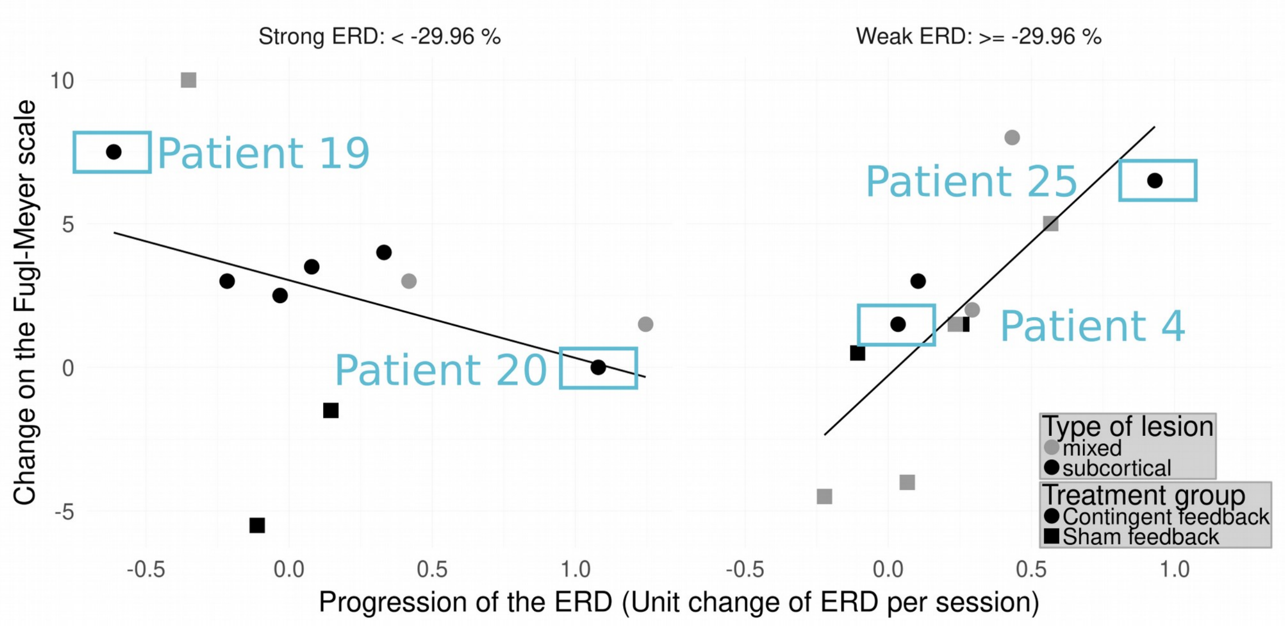

Figure S22

The time-frequency plots in figure S23 are presented for the patients marked here. This figure is otherwise identical to figure 2 of the main document.

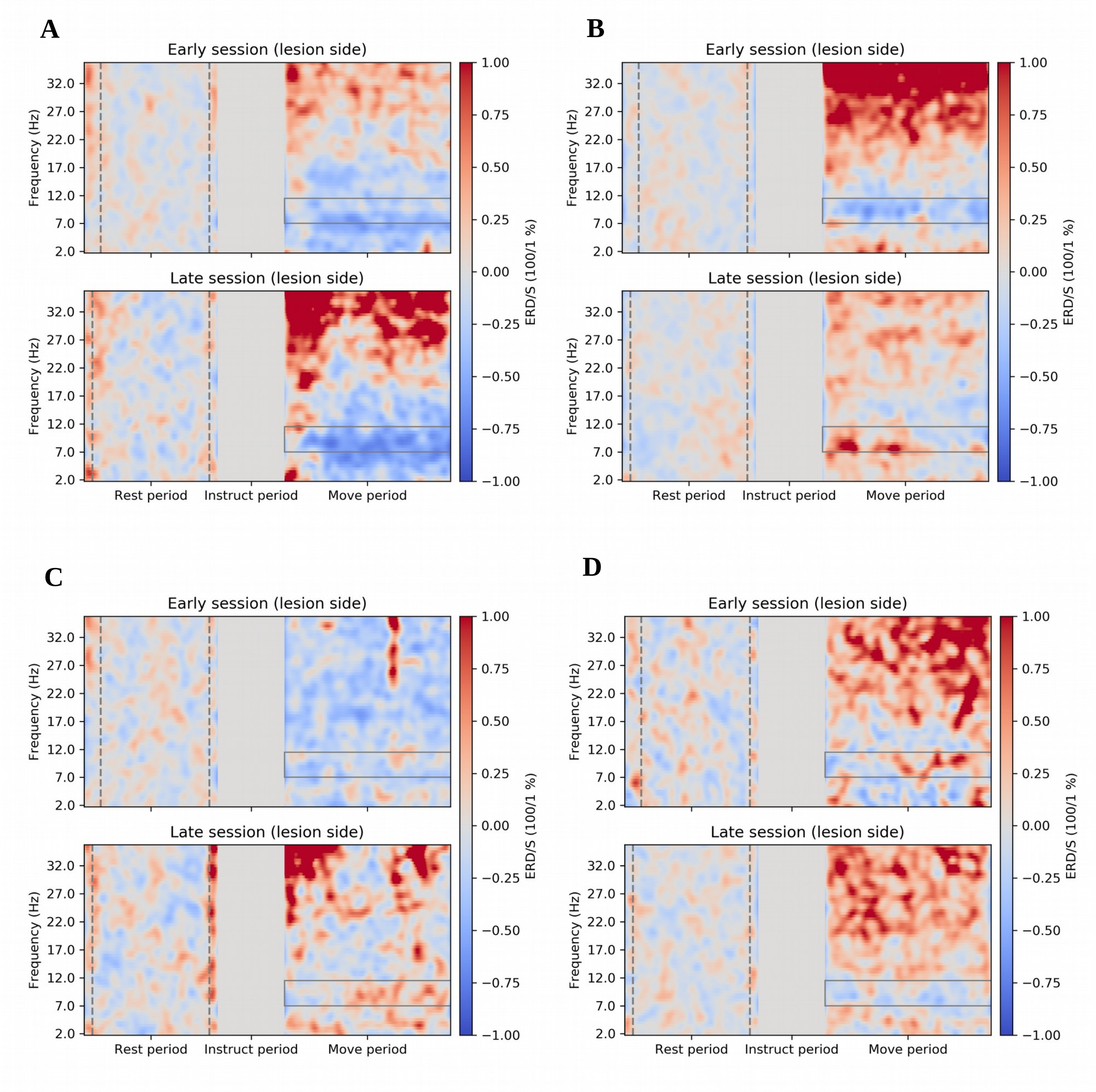

Figure S23: Time-frequency plots of sessions early and late in the training

Activity from all channels of interest on the hemisphere of the lesion has been averaged (i.e. average of channels C3, CP3, P3 or average of channels C4, CP4, P4). The duration of the *Rest* period is four seconds. The duration of the *Move* period is five seconds. The *Instruct* period has been disregarded in the analysis and is shown in light grey. The grey dashed lines mark the interval that was used to compute the baseline for ERD/S. The rectangle with the grey outline in the *Move* period shows the time and frequency range that was used to compute the ERD/S values.

A) Patient 19: Alpha desynchronization is stronger in the late session (sessions 1 and 16 out of 18 retained sessions presented)

B) Patient 20: Alpha desynchronization is weaker in the late session (sessions 1 and 11 out of 11 retained sessions presented)

C) Patient 25: Alpha desynchronization is weaker in the late session (sessions 1 and 11 out of 14 retained sessions presented)

D) Patient 4: Alpha desynchronization did not change on average (sessions 2 and 8 out of 9 retained sessions presented)

References

Park, W., Kwon, G. H., Kim, Y.-H., Lee, J.-H., & Kim, L. (2016). EEG response varies with lesion location in patients with chronic stroke. *Journal of NeuroEngineering and Rehabilitation*, *13*, 21. https://doi.org/DOI 10.1186/s12984-016-0120-2

Ramos-Murguialday, Ander, Broetz, D., Rea, M., Läer, L., Yilmaz, O., Brasil, F., … Birbaumer, N. (2013). Brain-machine interface in chronic stroke rehabilitation: a controlled study. *Annals of Neurology*, *74*(1), 100–108. https://doi.org/doi: 10.1002/ana.23879

Ray, A. M., Lopez-Larraz, E., Figueiredo, T., Birbaumer, N., & Ramos-Murguialday, A. (2017). Movement-related brain oscillations vary with lesion location in severely paralyzed chronic stroke patients. *Proceedings of the Engineering in Medicine and Biology Conference 2017*.

Van der Meer T, Te Grotenhuis M, Pelzer B. (2010). Influential Cases in Multilevel Modeling: A Methodological Comment. *Am Sociol Rev,* 75(1):173–8.
